# Impoundments facilitate upstream invasion and introgression: Case studies of fluvial black basses (*Micropterus* spp.) in the southeastern USA

**DOI:** 10.1371/journal.pone.0315620

**Published:** 2025-02-05

**Authors:** Andrew T. Taylor, Michael D. Tringali, James M. Long

**Affiliations:** 1 Department of Natural Resource Ecology and Management, Oklahoma State University, Stillwater, Oklahoma, United States of America; 2 Department of Biology, University of North Georgia, Dahlonega, Georgia, United States of America; 3 Florida Fish and Wildlife Conservation Commission, Fish and Wildlife Research Institute, St. Petersburg, Florida, United States of America; 4 U.S. Geological Survey, Oklahoma Cooperative Fish and Wildlife Research Unit, Department of Natural Resource Ecology and Management, Oklahoma State University, Stillwater, Oklahoma, United States of America; Albany Museum, SOUTH AFRICA

## Abstract

Impoundment construction has resulted in the alternation and loss of fluvial habitats, threatening the persistence of many native fishes. Compounding this threat, non-native species stocked into impoundments often invade interconnected fluvial habitats, where they may negatively affect native species. Black basses (genus *Micropteru*s) are popular sportfishes with divergent ecologies: some taxa are tolerant of impoundments and widely stocked to create fishing opportunities, whereas others are endemic fluvial specialists that are threatened by introgression with non-native congeneric taxa. We investigated whether impoundments facilitate non-native invasion and introgression in two case study systems: Lake Lanier, Georgia, and Lake Tenkiller, Oklahoma. In both case studies, native fluvial taxa inhabited upstream tributaries and a non-native was established within the downstream impoundment. Results from longitudinal surveys of upstream tributaries provided clear evidence that non-natives invaded upstream from impoundments, and in some cases, extensive introgression with native taxa also occurred. Variation in spatial trends of invasion and directionalities of introgression across case studies provided insights into eco-evolutionary drivers. Within the riverscapes studied, proximity to impoundment appeared to influence invasion and introgression dynamics, and in one case, stream size was also influential. Introgression rates also varied markedly across the species pairs studied–from very little introgression to the onset of hybrid swarming–illustrating the importance of underlying eco-evolutionary mechanisms such as habitat alteration, propagule pressure, and reproductive isolation. Our results underscore the need to consider the upstream influences of impoundments, and the non-natives that invade from them, to create more holistic riverscape conservation plans for fluvial fishes, including native black basses.

## Introduction

Anthropogenic alteration of landscapes has resulted in unprecedented imperilment of North America’s native fish fauna, particularly for stream fishes [1–3]. Primary threats to fluvial fish conservation include widespread alteration and loss of lotic habitats, fragmentation of populations, and introduction of non-native and invasive species [1, 3]. Understanding how these threats, and the interactions among them, influence species distributions, population declines, and extirpations within riverscapes is critical to informing conservation of fluvial fish biodiversity [4].

The construction of dams (i.e., barriers that impede streamflow) and creation of impoundments (i.e., lentic waterbodies) profoundly alters riverscapes. Downstream effects of dams are well-documented and include the interruption of nutrient and sediment transport, alteration of natural flow and temperature regimes, blockage of fish migrations, and loss of sensitive fish species [5–8]. However, impoundments also transmit disturbance upstream, affecting fishes inhabiting tributaries [9]. The effects of impoundments could synergistically drive native biodiversity loss in tributaries via habitat alteration, population fragmentation, and facilitating the invasion of non-native species. Habitat alteration resulting from the impoundment extends upstream within interface zones, which are dynamic in space and time as stream discharge and lake levels change, and typically feature excessive sedimentation and increased primary productivity [10]. Further, species that do not typically inhabit lentic habitats (i.e., obligate fluvial species) may become fragmented, resulting in elevated risk of inbreeding and local extirpation within remaining fluvial habitats in impoundment tributaries [11–13]. Impoundments also function as ‘stepping-stones’ of altered habitat within river networks, allowing invasion of interconnected fluvial habitats by non-native species (i.e. facultative fluvial species) that are often stocked within impoundments to provide increased sportfishing opportunities [14]. These upstream effects of impoundments can result in biodiversity loss and homogenized fish communities within interconnected fluvial habitats [15–18].

As both a source of ecological disturbance (i.e., habitat alteration) and propagule pressure (i.e., stocked non-native populations), impoundments influence outcomes of non-native species invasion and introgression events within riverscapes. Previous studies in terrestrial and aquatic ecosystems have demonstrated that the combined effects of disturbance and propagule pressure are often necessary for successful non-native species invasions [19–21]. Disturbance and propagule pressure are also important drivers of the speed and outcome of introgressive hybridization when considering closely-related fish species with weak reproductive isolation [22–24]. Both invasion and introgression can result in loss of native species inhabiting impoundment tributaries, either through competitive replacement, introgression of non-native alleles, or a combination of the two [22, 23]. When native populations in upstream tributaries are also fragmented by impoundments, they may become more susceptible to negative interactions with non-native invaders, including increased speed and severity of introgression [25, 26].

The black basses (Centrarchidae: *Micropterus*) provide an opportunity to examine the role of impoundments as vectors for non-native invasion and introgression with native, fluvial populations inhabiting upstream tributaries. Black basses are among the most popular sportfish species in the United States of America (USA), supporting a multi-billion dollar industry [27]. During an era of hydroelectric dam construction from the 1930’s through the 1970’s, large impoundments were created within river systems across the southeastern USA [27]. To bolster sportfishing opportunities in these impoundments, a number of species that persist in lentic habitats (i.e., facultative fluvial species) have been widely stocked outside their native ranges–both across neighboring basin boundaries and worldwide–including Largemouth Bass (*Micropterus nigricans*), Florida Bass (*Micropterus salmoides*, *formerly Micropterus floridanus*), Smallmouth Bass (*Micropterus dolomieu*), Spotted Bass (*Micropterus punctulatus*), and Alabama Bass (*Micropterus henshalli*). These introduced species tend to flourish in impounded habitats and often invade and establish into connected fluvial habitats.

In contrast, numerous black bass taxa are endemic to river drainages of the southeastern USA [28, 29], including Shoal Bass (*Micropterus cataractae*), Neosho Bass (*Micropterus velox*), Guadalupe Bass (*Micropterus treculii*), Chattahoochee Bass (*Micropterus chattahoochae*), and Bartram’s Bass (*Micropterus* sp. cf. *coosae*). These taxa are more specialized in their use of fluvial habitats than congener species stocked into impoundments; therefore, the damming of river systems across the southeastern USA has resulted in habitat loss for many endemic black basses with concomitant habitat expansion for non-natives stocked into impoundments [27, 30]. Non-native black basses threaten the conservation of endemic, fluvial taxa via the potential for interspecific competition and widespread introgression [30, 31]. Although sympatric black basses are generally thought to partition resources to reduce interspecific competition [32, 33], non-native black basses may overlap with natives in their diets and habitat use [34–36], suggesting interspecific competition could occur if resources were limited. Endemic black bass species are threatened by elevated rates of interspecific hybridization and introgression with non-native black bass species [31].

Outcomes from hybridization involving native and non-native black basses vary from hybridization without introgression, to widespread introgression, to complete admixture that results in the loss of parental species [31, 37]. Across many study organisms, it is commonly observed that first-filial (F_1_) generation hybrids may suffer no apparent loss of fitness, but second-filial (F_2_) generation hybrids and backcrosses may experience appreciable loss of fitness. Widespread backcrossing and admixture between endemic black basses and non-native congeners may degrade locally-adapted genomes, lead to the breakdown of co-adapted gene complexes, and allow for the incorporation of maladapted genes [38–41]. Within black basses, evidence of outbreeding depression in the form of increased disease susceptibility was documented in laboratory-produced F_2_ backcrosses between two distinct populations of Largemouth Bass [42]. Together, these mechanisms erode local genetic diversity and weaken the capacity to respond to global change stressors at both population- and species- levels [38, 43].

Extensive introgression and complete replacement of native black basses have been most commonly documented within impounded habitats following the introduction of a non-native species, with interspecific competition and selection pressures (natural or artificial) implicated as underlying drivers that favored non-native genomes [44–49]. The high abundance of non-native congeners in impoundments may also transmit propagule pressure that encourages invasion and introgression in upstream tributaries [50], which would threaten native fluvial species. Few studies have directly addressed the spread of non-native black bass from impoundments and the effects on recipient fluvial populations, though several studies have alluded to such situations across the southeastern USA [51–53]. Recent studies of native Bartram’s Bass in fluvial habitats of the Savannah River basin found that increased distance from impoundments, increased watershed forest cover, and increased stream gradient were associated with fewer hybrids with non-native Alabama Bass [54, 55]. Given that impoundments and their fisheries are fixtures within human-modified riverscapes [10], additional investigation of the upstream effects of impoundments on native black bass species is warranted.

Herein, we explore whether impoundments facilitate non-native invasion and introgression with native, fluvial black bass populations in two impounded systems in the southeastern USA. We combine longitudinal sampling of impoundment tributaries with population genetic analyses to examine spatial trends in the invasion of non-native species and introgression dynamics with native populations inhabiting impoundment tributaries. ***Case Study I*** was conducted on Lake Sidney Lanier, Georgia, (hereafter, Lake Lanier), and primarily involved native Shoal Bass and non-native Alabama Bass. ***Case Study II*** was conducted on Tenkiller Ferry Lake, Oklahoma, (hereafter, Lake Tenkiller), and involved native Neosho Bass and non-native Smallmouth Bass. For comparative purposes, we treated data collection and analysis of each case study similarly. We conclude with a discussion that synthesizes results within an eco-evolutionary framework and infuses relevant literature.

## Materials and methods

### Study areas and sample collections

#### Case Study I

**Fig 1:**
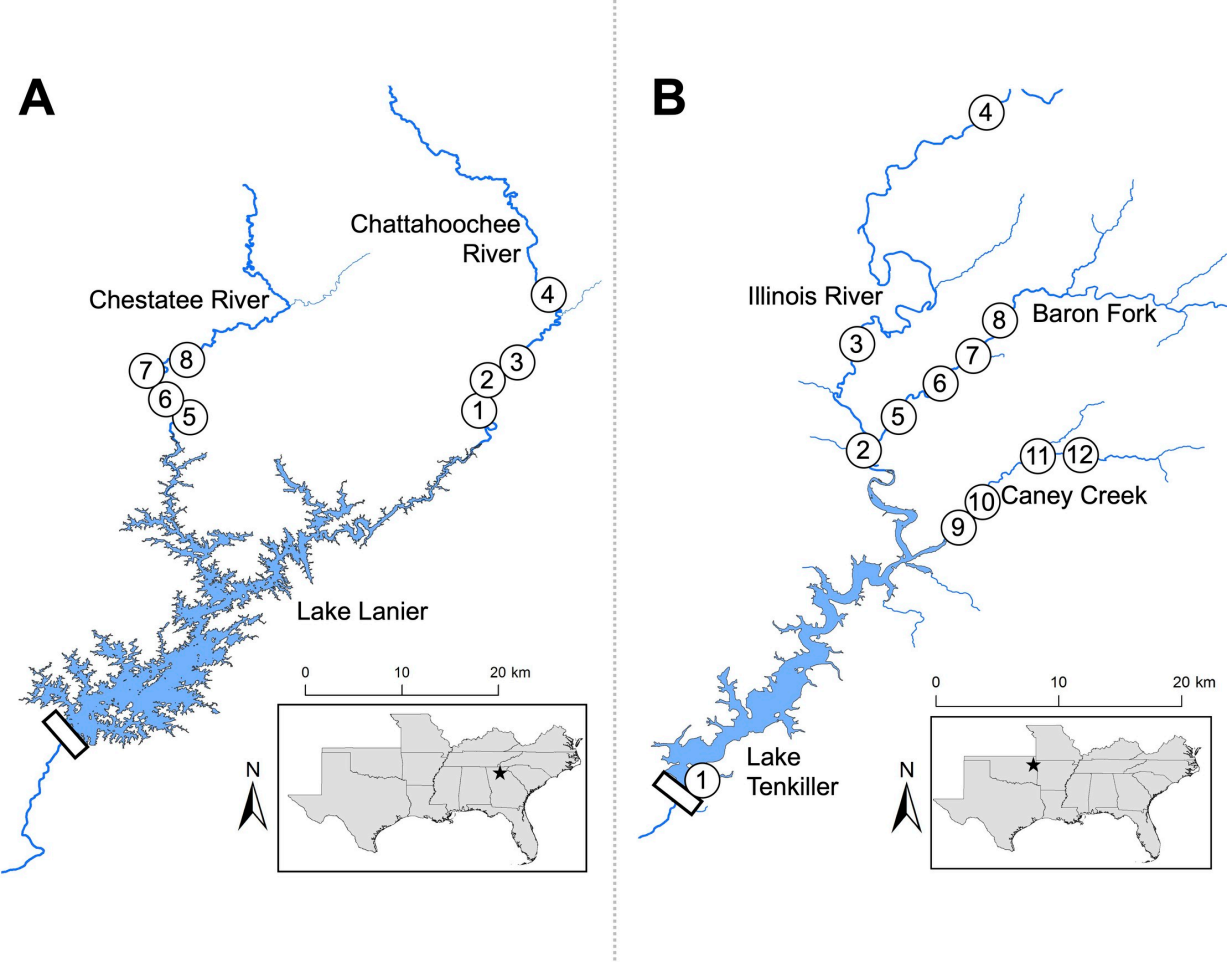
Study area and longitudinal sampling sites for A) *Case Study I* in Lake Lanier and B) *Case Study II* in Lake Tenkiller.

Lake Lanier, which was completed and filled by 1958 with a surface area of 150 km^2^, occurs within the upper Chattahoochee River basin, Georgia, and impounds the Chattahoochee and Chestatee rivers (S1 Table in S2 File, Fig 1A). The Chattahoochee River is a fifth-order tributary draining approximately 970 km^2^ as it enters the northeast portion of Lake Lanier, whereas the Chestatee River enters the lake’s northwest corner as a fourth-order stream that drains approximately 600 km^2^. The most direct route between the two river interfaces spans approximately 65 km of impounded habitat within Lake Lanier. Shoal Bass, a fluvial-specialist species native to the basin, inhabits the high-gradient shoal habitats that exist in both tributary rivers, but do not typically occupy or persist in impounded habitats [56–58]. Another native fluvial species, the Chattahoochee Bass, is often found in smaller, high-elevation tributaries and headwater reaches of mainstem rivers [59, 60]. In contrast, the non-native Alabama Bass was first documented in the Chestatee River near Lake Lanier in 1970, likely originating from unauthorized angler introduction(s) [56, 61, 62]. Alabama Bass in Lake Lanier currently support a popular sport fishery that has probably motivated additional unauthorized introductions of the species into drainages across the southeastern USA [45, 48, 53]. A previous study suggested that Shoal Bass in the Chattahoochee River upstream of Lake Lanier were relatively pure of non-native alleles [63], but introgression between Shoal Bass and non-native congeners represents a primary threat to Shoal Bass conservation throughout its native range [63–65], including areas farther downstream in the Chattahoochee River basin [66]. Native Chattahoochee Bass also appear to be threatened by introgression with non-natives, yet there are no published investigations to date [59, 60].

For the Lake Lanier study area, we used a jet-drive boat electrofisher to sample black bass at a series of four sites in each river that began at each river’s interface with Lake Lanier. Each sample site encompassed approximately 350 m of stream length, sampling effort was standardized to 15 min of pedal time at each site, and sample numbers represented raw catch of each species at each site. Most sample sites were situated near shoal habitats, but we also sampled surrounding riffles, runs, and pools. We collected fin-clips in May 2013 and May 2014 from all black bass excluding those immediately identified as Largemouth Bass, a relatively ubiquitous native species in the system. Field identifications were made based on a key that included morphology and coloration [66]. Collections were performed under the auspices of Oklahoma State University’s Institutional Animal Care and Use Committee’s protocol # AG-13-8 and a scientific collection permit issued by the Georgia Department of Natural Resources.

#### Case Study II

Lake Tenkiller was completed in 1952–53 within the Illinois River basin of Arkansas and Oklahoma with a surface area of 52 km^2^ (S2 Table in S2 File, Fig 1B). This impoundment is fed by two major tributary streams, the sixth-order Illinois River that drains approximately 2,529 km^2^ and the fifth-order Baron Fork that drains 896 km^2^ prior to its confluence with the Illinois River, situated just upstream of Lake Tenkiller. A smaller, fourth-order tributary, Caney Creek, drains 238 km^2^ prior to emptying into Lake Tenkiller east of the Illinois River confluence. Approximately 15 km of impounded habitat is situated between the interfaces of the Illinois River and Caney Creek. Neosho Bass, which were formerly considered a subspecies of Smallmouth Bass, were recently elevated to species status [29, 67]. Neosho Bass are native to all three tributaries to Lake Tenkiller; however, this species did not persist in impoundments constructed within its range [67, 68]. As a result, the stocking of non-native Smallmouth Bass into impoundments has been favored in the region. In 1991 and 1992, the Oklahoma Department of Wildlife Conservation (ODWC) stocked Lake Tenkiller with Smallmouth Bass (colloquially, the “Tennessee lake-strain” Smallmouth Bass) originating from Percy Priest Lake, Tennessee [51, 69]. A post-stocking survey in 1999 revealed that Smallmouth Bass in Lake Tenkiller had 85–90% non-native alleles, but no non-native alleles were detected upstream in the Baron Fork [69]. Researchers had previously warned that non-native alleles could quickly infiltrate native populations because Neosho Bass are restricted to small streams with more dynamic disturbance regimes [68, 69]. Recent broad-scale surveys have confirmed hybridization between Neosho Bass and Smallmouth Bass in the Illinois River upstream of Lake Tenkiller [70–73].

In May through September of 2015, we sampled black bass from the Illinois River (three sites), Baron Fork (four sites), and Caney Creek (four sites), wherein sampling sites spanned upstream from each system’s interface. We also sampled an additional site within Lake Tenkiller in 2014, focusing effort near the dam where non-native Smallmouth Bass were originally stocked. Sampling was conducted with boat- and backpack- mounted electrofishing and supplemented by angling in areas where electrofishing was impractical. Stream sample sites encompassed approximately 300 m of stream length, and we haphazardly sampled all available habitat types at each. We also set sample size targets that varied based on stream size, from 25 to 50 fish per site in the Illinois River, to 12 to 25 per site in Baron Fork and Caney Creek. Most collections at a site were completed within one day, but some required multiple visits to meet sample size targets. We fin-clipped all fish identified as Neosho Bass, Smallmouth Bass, or their hybrids in the field, but excluded specimens identified morphologically as native Largemouth Bass or Spotted Bass. Collections were performed under the auspices of Oklahoma State University’s Institutional Animal Care and Use Committee’s protocol # AG-13-8 and a scientific collection permit issued by the Oklahoma Department of Wildlife Conservation.

### Genotyping

We genotyped *Case Study I* samples with 16 di-nucleotide microsatellite markers developed to amplify in black bass species (*Msaf 05*, *06*, *08*, *09*, *10*, *12*, *13*, *17*, *22*, *24*, *25*, *27*, *28*, *29*, *31*, and *32*; [74]). Samples from *Case Study II* were genotyped using seven di-nucleotide microsatellite DNA markers developed to amplify in Smallmouth Bass and other black bass species (*Mdo*03, [75]; *Msaf* 01, 05, 06, 14, 17, and 29 [74]). Previous research has demonstrated the capability of both marker sets to diagnose introgression and population structure of black bass taxa, including specifics on DNA extraction, PCR amplification, and quality assurance [70, 76]. Prior to data analyses, we screened for duplicate genotypes using the multilocus matching function in GenAlEx v. 6.502 [77] and retained only the first collection of any duplicated genotype. Resulting genotypes are provided as Supporting Information for *Case Study I* (S1 Appendix) and *Case Study II* (S2 Appendix).

### Data analyses

To assess the invasion of non-native parental species, as well as introgression dynamics at each sample location, we employed an analytical workflow that combined genetic structure estimates and hybrid classes assignments, which were compared with one another along with hybrid index estimates. We also investigated the assignment power of each marker set to distinguish among parental species pairs experiencing hybridization. In each system, we summarized hybridization at individual- and site- levels to describe introgression dynamics [37].

#### Genetic structure

We examined the taxonomic composition of sampled individuals using the Bayesian clustering algorithm STRUCTURE 2.3.4 [78]. In STRUCTURE, the number of genetic clusters (*K*) can be heuristically estimated for a sample of individuals having unknown ancestry [79]. However, because we wanted to limit assignments of our unknown genotypes to nominal genetic clusters (i.e., clusters representing known black bass species) that were potentially present in our respective study systems, we genotyped DNA from selected reference specimens and included those genotypes on a system-specific basis, setting values of *K* accordingly (*Case Study I*, *K* = 5; *Case Study II*, *K* = 3). The selected reference specimens, whose genotypes provided the basis for individual assignment to nominal genetic clusters, were authenticated in previous studies [70, 76, 80]. Prior to adopting a final value of *K*, we first explored model runs with *K*– 1 and *K* + 1 for each system to ensure that no additional taxonomic signals existed within our unknown samples [70, 76].

For all runs of STRUCTURE, we implemented the admixture model with independent allele frequencies among taxa, using a burn-in of 50,000 and 1,000,000 MCMC repetitions. Ten independent model runs were conducted at each of the specified *K* values. Due to uncertainty regarding the origins of introduced taxa, we did not adopt the ‘PopFlag’ option, which would have restricted the assignment of our unknown samples based on allelic frequencies within the reference genotypes [78]. To produce a final, optimal alignment of independent STRUCTURE runs, we used STRUCTURE HARVESTER web v. 0.6.94 [81] and CLUMPP v. 1.1.2 [82] to perform cluster matching and permutation with the G′ pairwise matrix similarity statistic and the “LargeKGreedy” algorithm for 1,000 randomly sequenced runs. Finally, we plotted individual proportional assignments (*q*) to each taxonomic cluster to visualize hybrid identity, and we summarized spatial trends by aggregating *q* by sampling location to provide a coarse estimate of the overall genomic proportion (*Q*) of alleles assigned to each genetic cluster.

#### Hybrid classification

We assigned individual genotypes to hybrid class using the Bayesian algorithm in NEWHYBRIDS [83] as implemented in the ‘hybriddetective’ [84] and ‘paralellnewhybrid’ [85] packages for the R statistical computing language [86]. We assigned individuals to one of the following six classes: parental species one (P_1_), parental species two (P_2_), first filial hybrid (F_1_), second filial hybrid (F_2_), backcross to parental species one (BC_1_), and backcross to parental species two (BC_2_). NEWHYBRIDS estimates the posterior probability of an individual genotype belonging to each modeled hybrid class assuming two parental taxa are involved [83]; therefore, we used STRUCTURE results to delineate taxonomic pairs of interest. From reference allele frequencies, genotypes of parental species (*n* = 50 of each) were simulated using the “freqbasedsim_GTFreq” function. We designated non-hybrid parental genotypes during modeling (i.e., “Zeds”), which can improve the ability of NEWHYBRIDS to accurately model the expected genotypes of potential mixtures [83]. We ran three independent simulations of NEWHYBRIDS, each with a 500,000 burn-in length and 1,000,000 MCMC sweeps, then calculated mean posterior probabilities for class assignments across simulations. Posterior probability cutoffs for assignment to hybrid classes were determined following evaluation in ‘hybriddetective’ (see Assignment power). We also aggregated hybrid classifications at the site-level to report trends in proportion of hybrid individuals (*H*).

#### Hybrid index

Although STRUCTURE is useful in visualizing and detecting admixture within complicated systems, model complexity may hinder accuracy of admixture estimates [87]. Therefore, we corroborated our results from both STRUCTURE and NEWHYBRIDS with maximum likelihood-based estimates of individual hybrid index calculated in the ‘introgress’ R package [88]. We limited analyses to relevant taxa based on clusters in STRUCTURE that showed genotype admixture in the respective study system. Hybrid index values can range from 0 to 1, with extremum values representing either parental species and intermediate values signaling interspecific hybridization. We also computed interspecific heterozygosity with the “calc.intersp.het” function in ‘introgress’, wherein higher values indicate more recent hybridization. We visualized ‘introgress’ results with triangle plots that displayed estimates of hybrid index plotted against interspecific heterozygosity.

#### Assignment power

We estimated the power of each full marker set to assign individuals to hybrid classes in each study system using the ‘hybriddetective’ R package and recommended workflow [84]. We again limited analysis to relevant pairings of parental taxa and used the corresponding reference allele frequencies to simulate genotypes belonging to the same six hybrid classes modeled in NEWHYBRIDS. For each modeled parental species pair, we used the “freqbasedsim_GTFreq” function to generate two simulations of three replicates, wherein each replicate contained 50 simulated genotypes of each hybrid class. Then, for each simulated genotype, we estimated the posterior probability of belonging to each hybrid class using the Bayesian algorithm NEWHYBRIDS [83] with a burn-in of 50,000 and 1,000,000 Markov chain Monte Carlo (MCMC) sweeps. We evaluated accuracy and power of assignments to each hybrid class at three posterior probability cutoffs (0.50, 0.75, and 0.90).

## Results

### Case Study I

#### Collection summary

A total of 223 individuals were collected and genotyped from four sites in each tributary river, with sample sites spanning 6–24 river-kilometers (rkm) upstream of Lake Lanier in the Chattahoochee River and from 3–15 rkm upstream in the Chestatee River (Fig 1A). Based on putative field identifications, similar numbers of Shoal Bass were obtained in the Chattahoochee and Chestatee rivers, whereas Alabama Bass appeared more prevalent in the Chestatee River. Phenotypic Chattahoochee Bass and putative hybrids with Alabama Bass were encountered at the farthest upstream site on the Chattahoochee River. We also encountered phenotypic Redeye Bass (*Micropterus coosae*) in the Chestatee River–a species previously undocumented in the study area. Therefore, we included Chattahoochee Bass and Redeye Bass reference genotypes within our taxonomic assignments to corroborate field identifications.

#### Genetic structure

**Fig 2:**
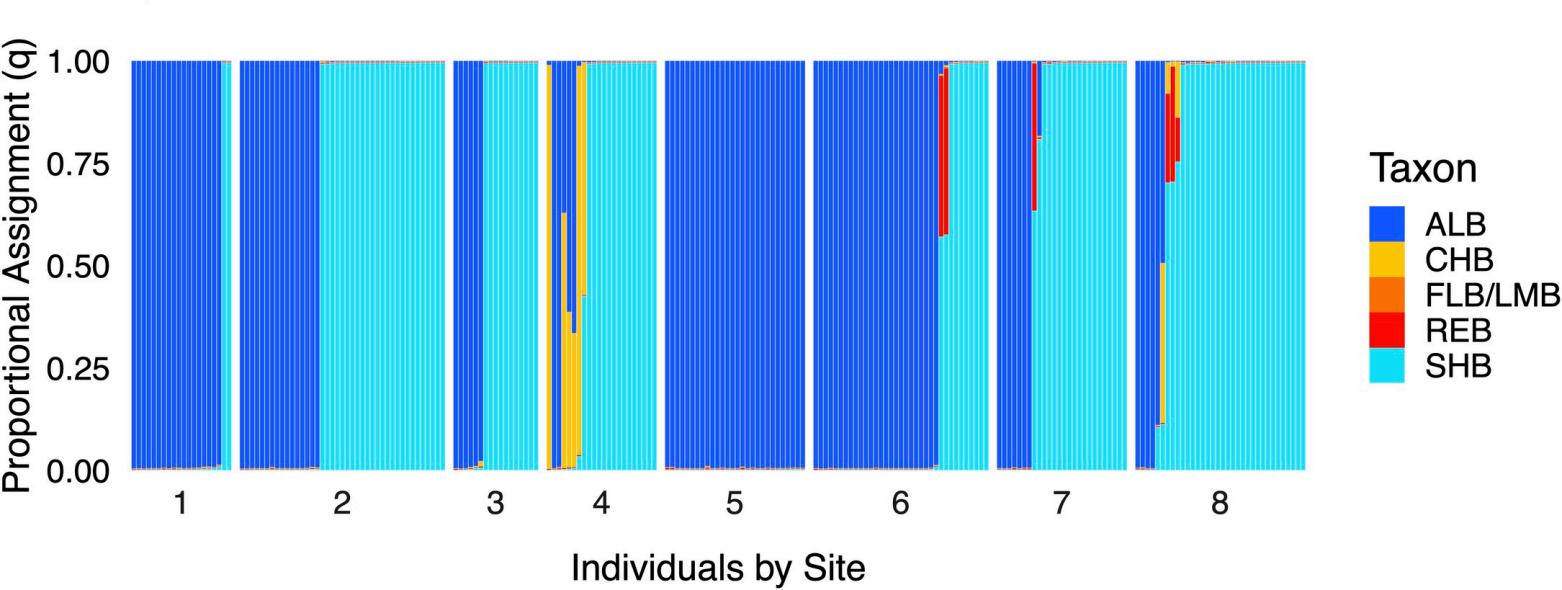
Individual STRUCTURE proportional assignments (*q*) for *Case Study I* (Lake Lanier, Georgia), ordered by sampling site. Cluster colors correspond to the following black bass taxa: non-native Alabama Bass (ALB), native Chattahoochee Bass (CHB), native Florida Bass or Largemouth Bass (FLB/LMB), previously undocumented Redeye Bass (REB), and native Shoal Bass (SHB).

**Fig 3:**
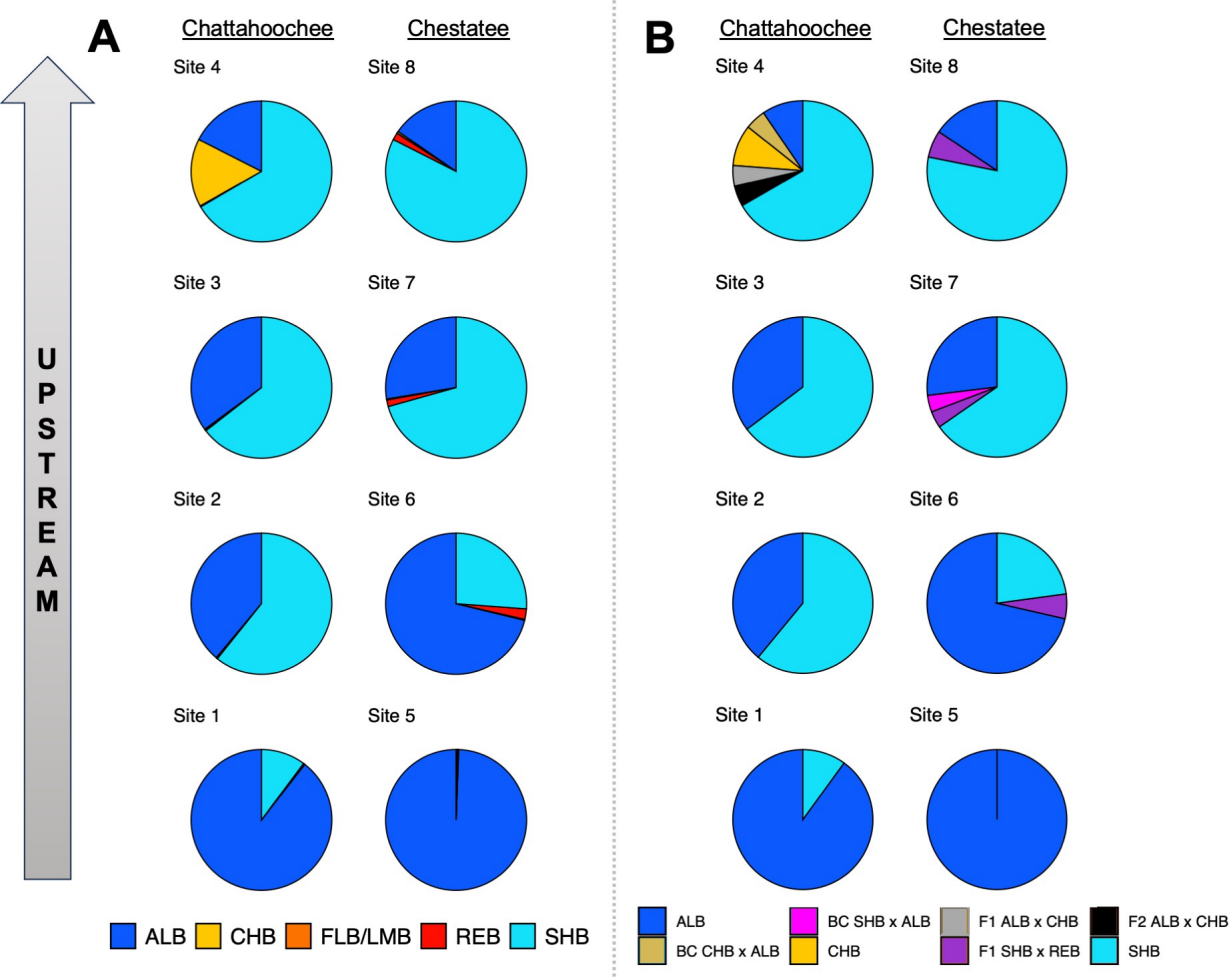
*Case Study I* (Lake Lanier, Georgia) site-level summaries of A) genomic proportions from STRUCTURE and B) hybrid classifications from NEWHYBRIDS. Cluster colors correspond to the following black bass taxa: non-native Alabama Bass (ALB), native Chattahoochee Bass (CHB), native Florida Bass or Largemouth Bass (FLB/LMB), previously undocumented Redeye Bass (REB), native Shoal Bass (SHB). Hybrid classes include backcrosses (BC), first filial hybrids (F_1_), and second filial hybrids (F_2_).

STRUCTURE assignments revealed three pairs of parental taxa producing hybrids: Shoal Bass x Alabama Bass, Chattahoochee Bass x Alabama Bass, and Shoal Bass x Redeye Bass (Fig 2, S1 Fig in S1 File). Non-native Alabama Bass were present at the uppermost sites in both tributaries, yet admixture with Shoal Bass was only observed in one specimen. Conversely, Chattahoochee Bass were present in our uppermost site in the Chattahoochee River, where we detected admixture between this native species and non-native Alabama Bass. Redeye Bass and associated hybrids with Shoal Bass were limited to sites upstream of the impoundment interface on the Chestatee River. At the site level, non-native Alabama Bass *Q* was highest with proximity to impoundment in each tributary, whereas Shoal Bass *Q* concomitantly increased moving upstream (Fig 3A). Shoal Bass *Q* increased from 0.10 and 0.00 at the farthest downstream sites in the Chattahoochee and Chestatee rivers, respectively, to 0.67 and 0.82 at the farthest upstream sites. Conversely, Alabama Bass *Q* was highest at the farthest downstream sites (0.89 and 0.99, respectively) and was lowest at the farthest upstream sites (0.18 and 0.15). Native Chattahoochee Bass contributed *Q* = 0.16 in the farthest upstream Chattahoochee River site, but those signatures were otherwise rare. Redeye Bass comprised *Q* of approximately 0.02 at each of three most upstream sites in the Chestatee River.

#### Hybrid classification

**Fig 4:**
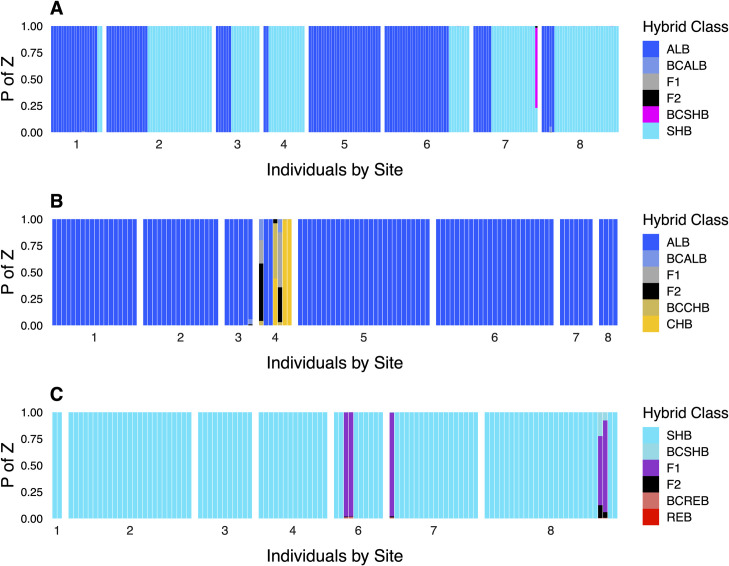
Individual NEWHYBRIDS results for *Case Study I* (Lake Lanier, Georgia) displaying estimated posterior probabilities of belonging to a given hybrid class (P of Z). Species pairings examined were: A) native Shoal Bass and non-native Alabama Bass; B) native Chattahoochee Bass and non-native Alabama Bass; and C) native Shoal Bass and previously undocumented Redeye Bass. Hybrid classes include backcrosses (BC), first filial hybrids (F_1_), and second filial hybrids (F_2_).

Individual hybrid class assignments in NEWHYBRIDS corresponded well with STRUCTURE results (Fig 4, S2 Fig in S1 File). Little evidence for hybridization or introgression amongst Shoal Bass and non-native Alabama Bass was recovered, except for a backcross towards Shoal Bass from Site 7 of the Chestatee River. We found the highest site-level *H* estimates were associated with upstream sites both tributaries; however, these findings were driven by unanticipated interactions with headwater-oriented species (Fig 3B). In the uppermost site in the Chattahoochee River (*H* = 0.14), three Chattahoochee Bass x Alabama Bass hybrids were recovered, including an F_1_, an F_2_, and a backcross towards Chattahoochee Bass. In the Chestatee River, we recovered F_1_ hybrids between Shoal Bass and Redeye Bass from Sites 6, 7, and 8 (wherein *H* varied from 0.06 to 0.08), but our sampling lacked non-hybrid Redeye Bass.

#### Hybrid index

**Fig 5:**
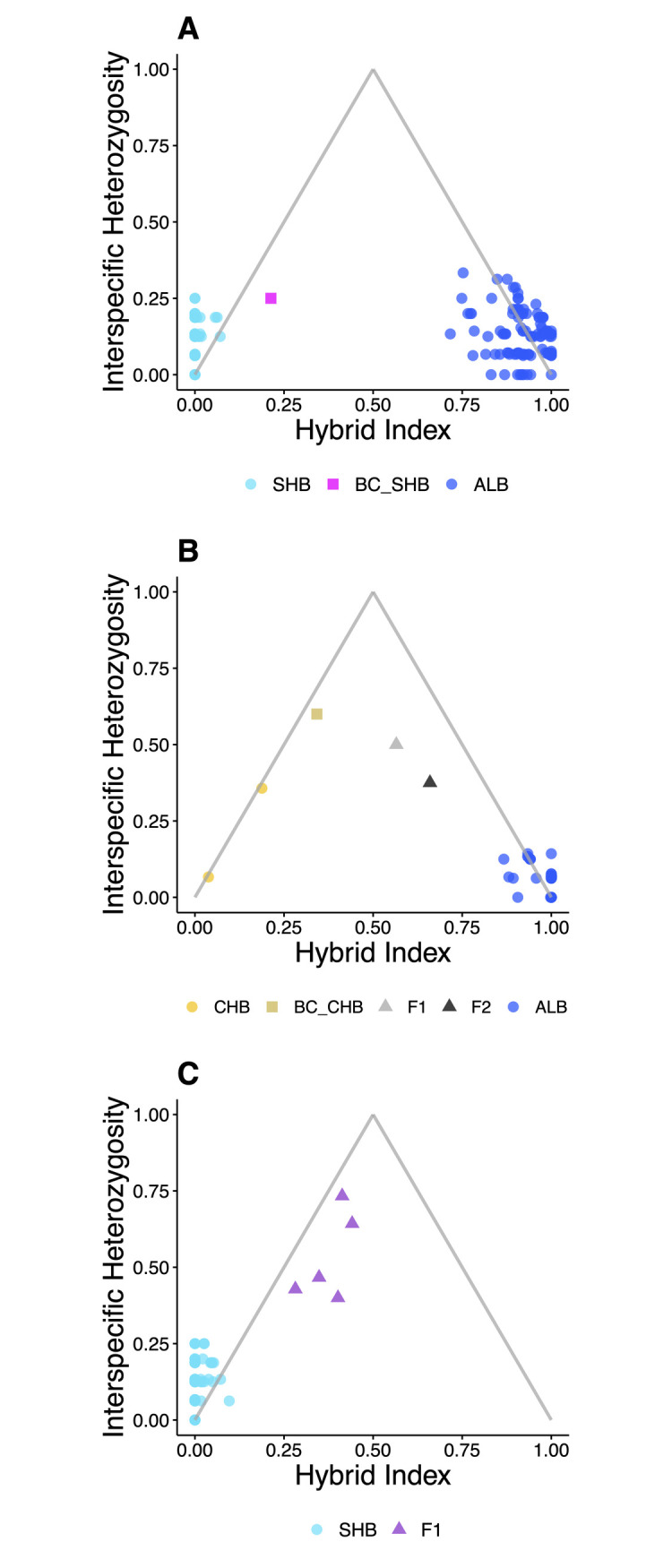
Triangle plots for *Case Study I* (Lake Lanier, Georgia) generated in ‘introgress’ that also display corresponding hybrid class assignments from NEWHYBRIDS. Species pairings examined were: A) native Shoal Bass and non-native Alabama Bass; B) native Chattahoochee Bass and non-native Alabama Bass; and C) native Shoal Bass and previously undocumented Redeye Bass. Shapes were used to contrast hybrid classifications, wherein circles represent non-hybrids, squares represent backcrosses (BC) and triangles represent both first filial (F_1_) and second filial (F_2_) hybrids.

Estimates of individual hybrid index and resulting triangle plots from ‘introgress’ largely corroborated assignments from STRUCTURE and NEWHYBRIDS (Fig 5). Fish associated with pure parentals of any taxon were characterized by low interspecific heterozygosity estimates and corresponding hybrid index values approaching zero or one. Individuals classified as hybrids by NEWHYBRIDS were typified by higher interspecific heterozygosity estimates and hybrid index estimates between approximately 0.25 and 0.75. Comparisons across methods revealed minor disagreements that could be attributed to differences in analytical methods, lack of diagnostic power, other sources of genotypic noise, or non-recency of admixture. For example, a specimen identified as an F_1_ Chattahoochee Bass x Alabama Bass did not display interspecific heterozygosity levels approaching one (signaling contemporaneous admixture), yet had a hybrid index estimate between 0.50 and 0.75.

#### Assignment power

Overall, simulation analyses indicated a high degree of accuracy and power to differentiate hybrid classes amongst the several parental species pairs of interest in Lake Lanier. Given this result, we chose an inclusive posterior probability cutoff of 0.50, accepting slightly lower assignment accuracies with the tradeoff of allowing all samples to be classified. Mean accuracy for Shoal Bass versus Alabama Bass was >0.95 for parental classes and >0.85 for all hybrid classes, and power estimates were ≥0.75 for all classes except F_2_ (= 0.65; S3 & S4 Figs in S1 File). Despite fewer reference genotypes for simulating admixtures involving either Chattahoochee Bass or Redeye Bass, results again suggested a high degree of accuracy (>0.85 for all classes) and power (≥0.75 for all classes, except F_2_ Chattahoochee Bass x Alabama Bass was 0.68; S5–S8 Figs in S1 File).

### Case Study II

#### Collection summary

A total of 268 individuals identified as Smallmouth Bass were collected and genotyped from 12 sites in the Lake Tenkiller study area: 1 site situated near Lake Tenkiller’s dam, 3 sites spanning 54 rkm upstream of the lake in the Illinois River, 4 sites spanning 19 rkm upstream in the Baron Fork, and 4 sites spanning 13 rkm upstream in Caney Creek (Fig 1B). In general, 12–30 individuals were collected at each site; however, a larger sample size (*n* = 47) was taken at the farthest upstream site on the Illinois River to coincide with a longer, 7.5 km sampling reach.

#### Genetic structure

**Fig 6:**
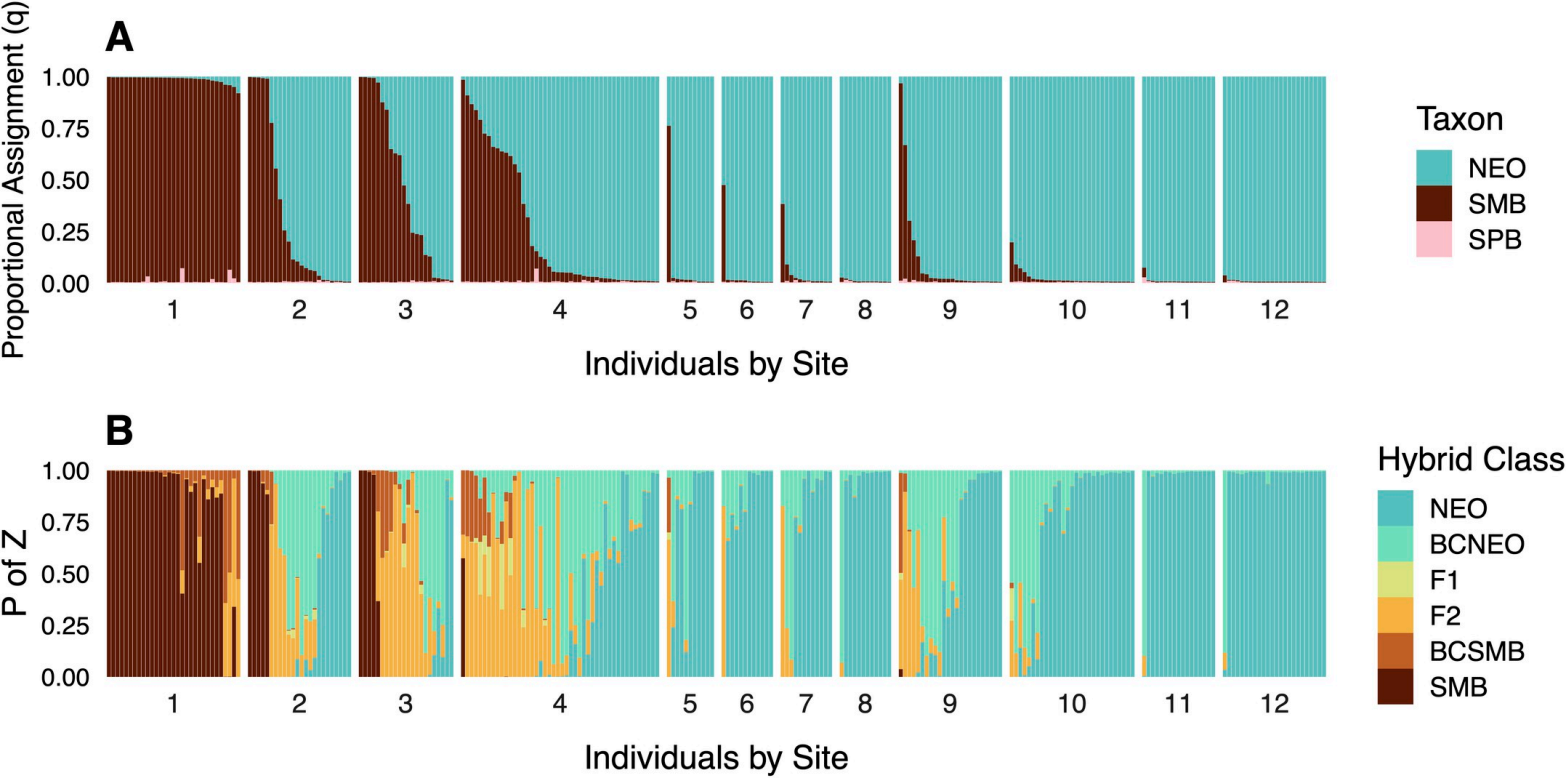
Individual assignments for *Case Study II* (Lake Tenkiller, Oklahoma), ordered by sampling site. Panels include: A) STRUCTURE proportional assignments wherein cluster colors correspond to native Neosho Bass (NEO), non-native Smallmouth Bass (SMB), and native Spotted Bass (SPB); and B) NEWHYBRIDS results displaying estimated posterior probabilities of belonging to a given hybrid class (P of Z) for mixtures of Neosho Bass and Smallmouth Bass. Note that discretizing hybrid classes at a critical posterior probability threshold of ≥0.50 resulted in some signal loss, such as backcrosses towards Smallmouth Bass (BCSMB) in Fig 7B. Hybrid classes include backcrosses (BC), first filial hybrids (F_1_), second filial hybrids (F_2_).

**Fig 7:**
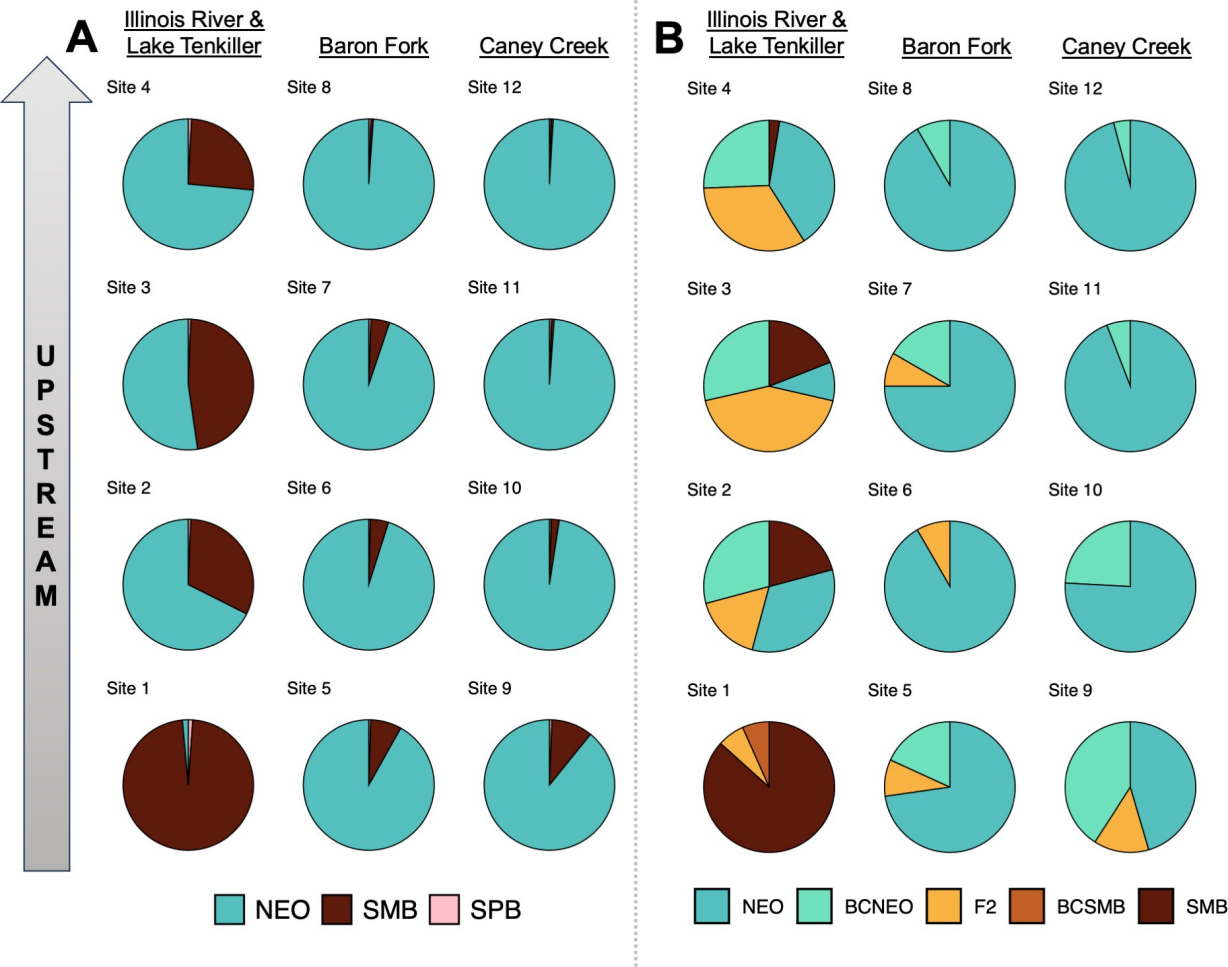
*Case Study II* (Lake Tenkiller, Oklahoma) site-level summaries of A) genomic proportions from STRUCTURE and B) hybrid classifications from NEWHYBRIDS. Black bass taxa and hybrid classes include: native Neosho Bass (NEO), non-native Smallmouth Bass (SMB), native Spotted Bass (SPB), backcrosses to parental species (BC), first filial hybrids (F_1_), and second filial hybrids (F_2_).

At the individual level, STRUCTURE assignments revealed considerable admixture between Neosho Bass and non-native Smallmouth Bass (Fig 6A, S9 Fig in S1 File). Low amounts of sympatric Spotted Bass ancestry were also assigned, particularly in fish with predominantly Smallmouth Bass ancestry in Lake Tenkiller and the Illinois River. At the site level, non-native Smallmouth Bass *Q* increased, and Neosho Smallmouth Bass *Q* concomitantly decreased, with proximity to impoundment in each tributary (Fig 7A). The site within Lake Tenkiller was dominated by Smallmouth Bass alleles (*Q* = 0.97), with very low presumptive contributions from Spotted Bass and Neosho Bass. In the smaller tributaries, Caney Creek and Baron Fork, Neosho Bass *Q* was relatively high, ranging from 0.89 at sites nearest impoundment to 0.99 at the farthest upstream sites. In contrast, the larger Illinois River had much higher levels of non-native ancestry, with Smallmouth Bass *Q* ranging from 0.26 to 0.47. Sympatric Spotted Bass alleles appeared to have low site-level contributions of *Q* ≤ 0.01 throughout, which could reflect noise as opposed to signal.

#### Hybrid classification

NEWHYBRIDS results were in good agreement with STRUCTURE results (Fig 6B, S10 Fig in S1 File). Non-hybrid Smallmouth Bass were recovered from Lake Tenkiller and all three sites on the Illinois River, but were absent from Baron Fork or Caney Creek. Substantial introgression was observed in all Illinois River sites, with Neosho Bass x Smallmouth Bass F_1_/F_2_ hybrids and backcrosses towards Neosho Bass comprising up to approximately half of individuals sampled at a given site. In Baron Fork and Caney Creek, sites closer to the impoundment had low proportions of Neosho Bass x Smallmouth Bass F_1_/F_2_ or backcrosses towards Neosho Bass. Across the study region, backcrossing was predominately towards native Neosho Bass, with only two backcrosses towards Smallmouth Bass observed. Even the farthest upstream sites in Baron Fork and Caney Creek–perhaps the most buffered from impoundment propagules–contained at least one backcross towards Neosho Bass (Fig 7B). In the two smaller tributaries, *H* trended as expected with proximity to impoundment, ranging from *H* ≤ 0.08 in the headwaters of both systems to maximums of 0.27 in Baron Fork and 0.55 in Caney Creek. In the Illinois River, however, there was no apparent longitudinal trend in *H*, which varied from 0.46 to 0.71.

#### Hybrid index

**Fig 8:**
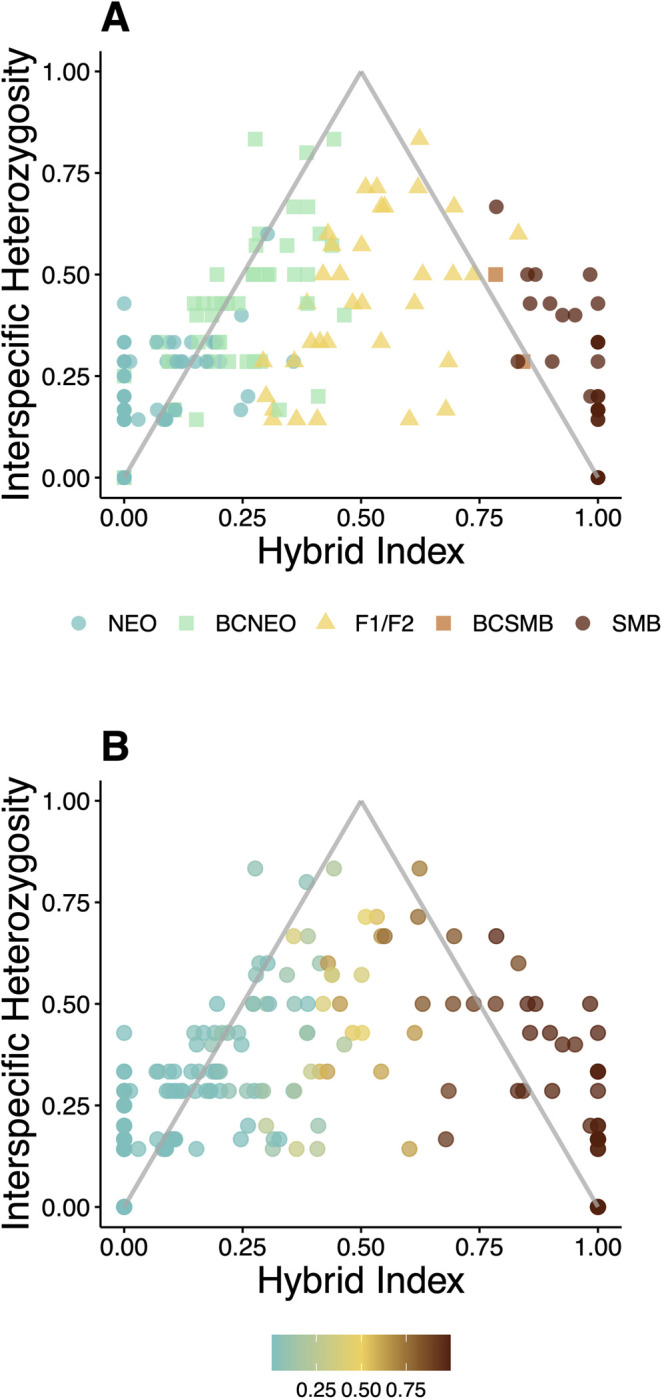
Triangle plots for *Case Study II* (Lake Tenkiller, Oklahoma) generated in ‘introgress’ that display patterns of introgression between native Neosho Bass (NEO) and non-native Smallmouth Bass (SMB). Panel A) displays corresponding hybrid class assignments from NEWHYBRIDS. Shapes were used to contrast hybrid classifications, wherein circles represent non-hybrids, squares represent backcrosses (BC) and triangles represent both first filial (F_1_) and second filial (F_2_) hybrids. Panel B) displays corresponding STRUCTURE proportional assignments (*q*) to non-native Smallmouth Bass (SMB).

Individual hybrid index estimates and resulting triangle plots largely corroborated assignments from STRUCTURE and NEWHYBRIDS, but also revealed some discrepancies (Fig 8). Comparisons across all three methods reaffirm a great deal of admixture has occurred between Neosho Bass and Smallmouth Bass. However, discrepancies in the marker-set sizes likely led to more assignment noise (see Assignment power). For example, interspecific heterozygosity estimates by ‘introgress’ were quite variable, whereas hybrid index estimates appeared more precise, for fish that the other two methods identified as non-hybrids.

#### Assignment power

Overall, simulation analyses indicated decent accuracy and moderate power to differentiate hybrid classes amongst Neosho Bass and Smallmouth Bass in Lake Tenkiller (S11 & S12 Figs in S1 File). We again chose the inclusive posterior probability cutoff of 0.50, accepting potentially lower accuracies with the tradeoff of allowing more samples to be classified. At this threshold, *n* = 11 genotypes were left unclassified (i.e., these fish lacked a hybrid class with ≥0.50 posterior probability). Mean accuracy was >0.80 for most classes, with the exception of F_2_ (0.66) and backcrosses towards Neosho Bass (0.75). Estimates of power varied across classes, with high power for parental classes (≥0.88), moderate power for F_1_ (0.75), and lower power for F_2_ (0.39) and backcrosses in either direction (0.48–0.53). For these reasons, we chose to combine F_1_ and F_2_ classes and took additional care in interpretation of hybrid class results.

## Discussion

These two case studies provide novel insights into how non-native black bass species introduced into impoundments can invade upstream tributaries and alter native black bass genomes, perhaps jeopardizing native population fitness and viability. In both systems, we documented that non-natives have spread upstream from impoundments into fluvial habitats, and in many cases, introgressive hybridization with native fluvial species has ensued. Here, we provide a synthesis of our two case studies, highlighting important differences between them. We do so within a simple eco-evolutionary framework that may facilitate the identification of primary drivers and the gauging of risks from non-native species invasions and introgressive hybridization for many similar (and often understudied) cases in impounded river systems across the southeastern USA. We conclude by discussing conservation implications of our findings and outlining future research needs.

### Eco-evolutionary synthesis

**Fig 9:**
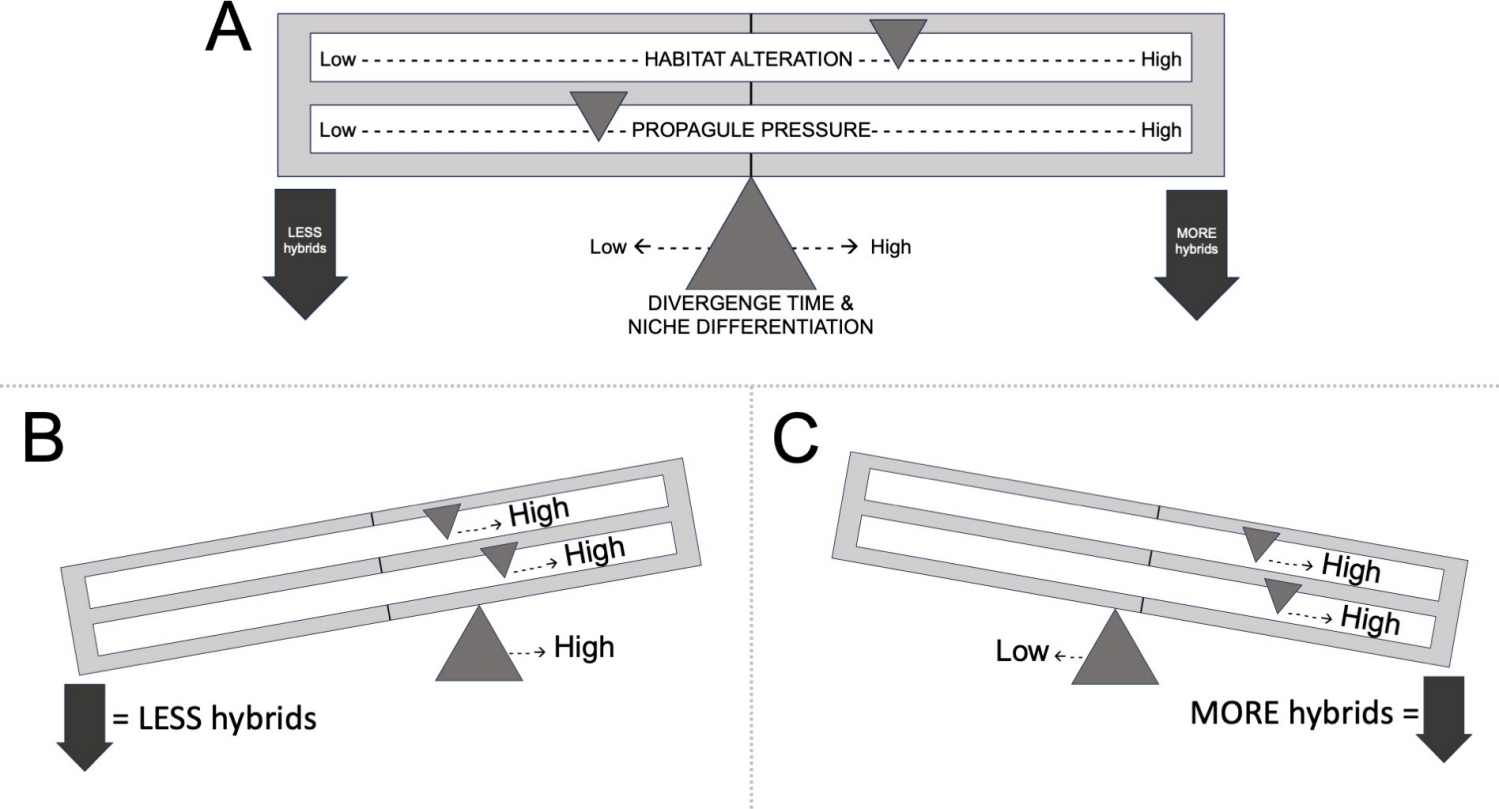
Our simple “TLT” (pronounced *tilt*) framework for contextualizing the eco-evolutionary drivers of invasion and introgression among black basses. Panel A) illustrates two sliding weights along a beam that toggle habitat alteration and propagule pressure levels, which can become elevated when near impoundments that often harbor non-native black basses. The fulcrum can also move slightly in either direction to: set the playing field” of interactions based on divergence time and ecological niche differentiation of the species in question. Bottom panels show B) an approximation of *Case Study I*’s (Lake Lanier, Georgia) pairing of native Shoal Bass and non-native Alabama Bass, and C) an approximation of *Case Study II*’s (Lake Tenkiller, Oklahoma) pairing of native Neosho Bass and non-native Smallmouth Bass.

Regarding the drivers of invasion and introgression, a large body of scientific literature focuses on a few broad factors that influence invasion success and introgression outcomes. Generally, success of invasions is expected to increase with greater ecological disturbance (i.e., habitat alteration) and with greater propagule pressure [89–91]. These two factors also are expected to influence introgression in similar ways, with elevated hybridization or introgression rates expected in areas of increased ecological disturbance and propagule pressure [92]. Outcomes of introgression can also be influenced by divergence time and ecological niche differentiation, with longer divergence times and greater degrees of niche differentiation leading to less frequent and less severe introgression [92–94]. For illustrative purposes, we simplify these eco-evolutionary drivers of invasion and introgression into an eponymous “TLT” (pronounced *tilt*) framework–a balance comprised of sliding weights and a moving fulcrum (Fig 9A)–that may aid in pinpointing ecological and species combinations that are more susceptible to introgression.

Once non-native black basses have invaded upstream habitats of fluvial endemic black basses, predicting the outcomes of hybridization and introgression becomes much more nuanced and difficult. As a general rule, ethological mating dynamics must be stabilized by some form of endogenous or exogenous post-fertilization selection against hybrids if parental taxa in admixed populations are to continue to evolve independently [95]. Postzygotic barriers to hybridization (reduced zygote viability or offspring fitness) are typically not effective at maintaining species boundaries across black bass taxa because of their relatively brief divergence times, which span from 7.39 MYA for the common ancestor of all black basses, to as recent as 0.68 MYA for more recently diverged species [29, 31, 96]. Therefore, prezygotic barriers–which may include ecological (e.g., different spawning habitats), behavioral (e.g., visual courtship), and temporal (e.g., different temperature cues for spawning activity) mechanisms–are essential to maintaining species boundaries in this genus [31, 96]. Black basses that have evolved in sympatry (e.g., Suwannee Bass, *Micropterus notius*, and Florida Bass) may have lower incidences of hybrids because they have had adequate time for niche differentiation that encourages prezygotic barriers; conversely, when non-natives are introduced into artificial sympatry, prezygotic barriers are typically weaker [31]. Prezygotic barriers can be altered or overwhelmed by the influences of habitat alteration and propagule pressure, which means that hybridization is often spatially and temporally dynamic, further complicating predictions of hybridization outcomes.

Both of our case studies, combined with other studies of black basses, demonstrate that impoundments facilitate invasion of non-native black basses into fluvial habitats by way of habitat alteration and creation of propagule pressure. In 2004, a survey of native Bartram’s Bass and non-native Alabama Bass in the Savannah River basin detected non-natives and hybrids at a few tributary sites nearest impoundments; however, only 5–6 years later, non-natives and their hybrids had quickly spread upstream and into many other tributaries [53]. More comprehensive evaluations revealed widespread hybridization in the Savannah River basin, but particularly in tributaries nearest to impoundments containing Alabama Bass [54, 55]. Similarly, non-native Florida Bass stocked into Texas impoundments have infiltrated native Largemouth Bass populations inhabiting upstream tributaries, with non-native haplotypes detected up to 80 km upstream of source impoundments [97]. Although the relative contributions of ecological disturbance versus propagule pressure remain unknown in these studies, in most situations it is likely a combination of both factors that leads to invasion success. The artificial and transitory habitats (e.g., water level fluctuations) created in river-impoundment interfaces are likely to differentially favor non-natives that are often characterized as habitat generalists [10, 98]. Couple those alterations with the propagule pressure generated by the establishment of non-native black bass fisheries in impoundments, and the successful upstream invasion of non-native black basses into fluvial habitats becomes a reasonable expected outcome in most cases [14, 50]. However, the outcomes of introgression can be quite varied, as our two case studies illustrate.

Our results for *Case Study I*, combined with an assessment from approximately 10 years prior [63], demonstrate that Alabama Bass have invaded far upstream in both tributaries, yet hybridization rates between Shoal Bass and non-native Alabama Bass may have been stable and low over that period. In 2005, 22 putative Shoal Bass were sampled in the Chattahoochee River upstream of Lake Lanier and 20 (91%) were considered non-hybrid Shoal Bass, with only one F_1_ hybrid (4.5%) and one backcrossed individual (4.5%) [63]. Propagule pressure of Alabama Bass moving upstream from an abundant population in Lake Lanier has likely occurred since at least the 1980’s, and fluvial habitats have been altered in both upstream tributaries–not only by the interface of the impoundment (where Alabama Bass were most abundant), but also by increased sedimentation and turbidity from historic mining and recent riparian development [99]. Lower hybridization rates between these species could be explained by a longer divergence time of 5.36 MYA to the most recent common ancestor [29], coupled with greater niche differentiation (Fig 9B). In this case, Shoal Bass may select faster-flowing shoal habitats to spawn in compared to Alabama Bass, which generally spawn in shallow littoral areas [62, 100]. At minimum, some ecological prezygotic barriers exist, if not also other behavioral or temporal barriers–but more ecological study could help identify such barriers and their interactions with the environment.

In the same system, *Case Study I* also revealed active introgression between native Chattahoochee Bass x non-native Alabama Bass in the farthest upstream site on the Chattahoochee River. Chattahoochee Bass have less divergence time with Alabama Bass (3.79 MYA to most recent common ancestor [29]) and niche differentiation and prezygotic barriers could be weaker between this species pair–however, ecological study of the endemic Chattahoochee Bass is warranted to understand how prezygotic species boundaries are compromised. Low sample size limits our inferences, but efforts are underway to quantify hybrid dynamics of Chattahoochee Bass throughout the upper Chattahoochee River basin (Steven Patrick, University of Georgia Cooperative Extension, oral comm., 2023).

An unexpected result from *Case Study I* was the confirmed presence of Redeye Bass hybrids with Shoal Bass in the Chestatee River. Further investigation could help characterize the extant range of non-hybrid Redeye Bass, and associated hybrids with Shoal Bass, throughout the Chestatee River and its tributaries. Molecular approaches could help determine whether Redeye Bass entered the system naturally via a headwater stream capture with the neighboring Etowah River basin, or were potentially introduced by anglers–a question that has arisen elsewhere within the Redeye Bass species complex [101, 102].

Results from *Case Study II* revealed substantial introgression between native Neosho Bass and non-native Smallmouth Bass, which has progressed quickly from initial stocking of Lake Tenkiller in the early 1990’s. Following stocking of Smallmouth Bass, a genetic assessment 7–8 years afterwards revealed 85–90% non-native alleles in Lake Tenkiller, but no non-native alleles were detected upstream in the Baron Fork [75]. Approximately 23 years post-stocking, our study revealed that non-native Smallmouth Bass and their hybrids occur in upstream reaches and substantial introgression has altered Neosho Bass genomes. Propagule pressure has likely increased upstream over time as the stocked Smallmouth Bass population has expanded in Lake Tenkiller, and lower reaches of all three streams are altered by impoundment inundation during flooding. Couple these drivers with a much shorter divergence time between Neosho Bass and Smallmouth Bass (1.51 MYA to most recent common ancestor [29]), and less niche differentiation between these closely related species, and the eco-evolutionary drivers tilt towards extensive introgression (Fig 9C). Following our study, genetic monitoring of the system in 2019-2021confirmed that Smallmouth Bass and their hybrids have increased in prevalence and now appear as a hybrid swarm that is replacing native Neosho Bass in the Illinois River [103].

Despite this expectation in *Case Study II*, the number of invasives and the amount of introgression varied markedly across a spatial gradient of stream size. Small stream size appeared to favor Neosho Bass and limit invasion and introgression with Smallmouth Bass. For example, Caney Creek was the smallest stream we sampled and, despite its close proximity to initial stocking sites in Lake Tenkiller, we recovered very low proportions of non-native alleles. In contrast, results were particularly concerning in the largest stream–Illinois River–where non-hybrid Neosho Bass comprised less than half of sampled fish at all sites. The proximate eco-evolutionary mechanisms behind this finding remain uninvestigated, but a reasonable hypothesis is that Neosho Bass are better adapted to the environmental conditions within smaller streams than the non-native Smallmouth Bass, which originated from the larger Tennessee River system. Similar spatial and environmental factors, like elevation, water temperature, and migration barriers, have been found to counteract invasion and hybridization in other fishes [55, 104]. In contrast, Alabama Bass in *Case Study I* did not appear to experience similar limits on upstream invasion, underscoring the importance of species-specific ecology and life-history requirements in determining invasion and introgression outcomes [105].

Overall, the eventual outcome of invasion and introgression in each case study remains uncertain because of the spatially and temporally dynamic nature of prezygotic reproductive barriers. In general, the speed at which native species are lost to extinction by hybridization increases when: 1) reproductive barriers are weak between taxa; 2) the native species is relatively rare or low in abundance; and 3) the non-native species gains a competitive advantage over the native species [106]. Whether the propagule pressure applied by non-natives entering fluvial habitats from the impoundments increases, remains steady, or decreases over time is an important consideration, as increased propagule pressure could eventually overwhelm any opposing ecological mechanisms that favor natives [23]. Furthermore, environmental variability associated with fluvial habitats (i.e., dynamic streamflow and disturbances) may periodically cause poor recruitment of native populations in these habitats [107, 108], which could lower abundance of natives and lead to increased invasion success and hybridization. Although evidence for interspecific competition between black bass species is mostly indirect, native fluvial species are generally more specialized in their habitat use and diets than their non-native counterparts [35, 36]. Native specialist species may be able to maintain a stronghold over non-native species in unaltered habitats [55, 104], but alteration of habitats could erode selective pressures that favor natives against non-native invasion and introgression. Postzygotic isolating mechanisms like reduced fitness (i.e., outbreeding depression) of hybrids could favor the maintenance of species boundaries in some cases. For example, in a study on the Flint River, Georgia, the proportions of hybrid classes between native Shoal Bass and non-native Spotted Bass were lower than expected from random mating, suggestive of purifying selection [64]. However, hybrids that experience little to no reduction in fitness could completely swamp or displace the native species. Ultimately, these uncertainties underscore the importance of continued monitoring of invasion extent, hybridization rates, and introgression dynamics to ensure conservation of native fluvial species.

### Conservation implications and future directions

The results of these two case studies reveal existing, and potentially impending, consequences of impoundment construction and non-native species introductions on the conservation of native black bass diversity in riverscapes across the southeastern USA. Hybridization among sympatric black bass species is a natural phenomenon, but increased hybridization rates resulting from habitat alteration and introduction of non-native congeners is unnatural [31]. Habitat alteration and ensuing hybridization often create a final push towards extinction for many native fluvial fishes [2, 3]. In the case of native black bass conservation, once large populations of non-natives become established in impoundments, the resulting perpetual source of propagule pressure could create a problem that becomes too large and costly to justify conservation actions for native taxa. As such, concerted efforts to educate managers and anglers on the negative effects of non-native species introductions (e.g., [109]), along with the enforcement of strict invasive species laws, could help reduce the number of introductions that occur in the future. Once non-native black bass become established and begin invading stream habitats, stocking programs designed to swamp tributary populations with native genetics could temporarily alleviate hybridization concerns [110], but non-native propagule pressure originating from downstream impoundments will likely outlast and overwhelm short-term stocking efforts. Identifying genetic refuge populations above movement barriers or creating artificial refuge populations may also be prudent, particularly for endemic headwater species like the Chattahoochee Bass that may have few, if any, non-introgressed populations remaining. Managers could also prioritize measures to maintain relatively intact habitats and watersheds, as maintaining near-natural instream habitats and flow regimes may give native populations the best chance to persist alongside invaders in human-altered riverscapes [54, 55, 104, 111].

Additional research is warranted to inform native black bass conservation, in our two case study systems and broadly across impounded river systems of the southeastern USA. Many questions remain in our case study systems that will require continued genetic monitoring paired with ecological research. In *Case Study I*, could ongoing and future stream habitat alteration induce increased hybridization between Shoal Bass and Alabama Bass? In *Case Study II*, will Smallmouth Bass propagule pressure eventually swamp and replace native Neosho Bass in the Illinois River? Similarly, will conditions in the headwaters of smaller streams continue to selected against non-native genomes, or will this eventually be overcome by propagule pressure or habitat alteration? Beyond our case studies, additional studies at both broad and fine spatial scales could be beneficial. Broad scale studies could clarify the specific eco-evolutionary mechanisms that influence the spatial extent, dynamics, and long-term outcomes of invasion and introgression in black basses inhabiting impounded river systems. At finer scales, studies identifying what prezygotic ecological, behavioral, or temporal barriers may exist between natives and non-natives in a system could help to predict how introgression dynamics may change over time with habitat alteration or propagule pressure.

## Supporting information

S1 Appendix Genotypes for *Case Study I* in Lake Lanier, Georgia, in .xlsx format. (XLSX)

S2 Appendix Genotypes for *Case Study II* in Lake Tenkiller, Oklahoma, in. xlsx format. (XLSX)

S1 File S1 through S12 Figs in a single .pdf file. (PDF)

S2 File S1 and S2 Tables in a single .pdf file. (PDF)

S1 Text Input data files and R code to reproduce data analyses and visualizations are available on GitHub: https://github.com/ATaylorFish/Impoundments_Hybrids. (TXT)
