# Supplementary material for: Impoundments facilitate upstream invasion and introgression: Case studies of fluvial black basses (Micropterus spp.) in the southeastern USA
Source: PLoS One. 2025 Feb 5;20(2):e0315620. doi: 10.1371/journal.pone.0315620 (PMC11798496; doi:10.1371/journal.pone.0315620)
Supplement: S1 File — (PDF) [file pone.0315620.s003.pdf]

Impoundments facilitate upstream invasion and introgression:
Case studies of fluvial black basses (*Micropterus* spp.) in the southeastern USA

Andrew T. Taylor[1,2]*, Michael D. Tringali[3], and James M. Long[4]

[1] Department of Natural Resource Ecology and Management, Oklahoma State University, Stillwater, Oklahoma, USA. ORCID: 0000-0002-8491-9967

[2] Department of Biology, University of North Georgia, Dahlonega, Georgia, USA.

[3] Florida Fish and Wildlife Conservation Commission, Fish and Wildlife Research Institute, St. Petersburg, Florida, USA. ORCID: 0000-0002-9336-9207

[4] U.S. Geological Survey, Oklahoma Cooperative Fish and Wildlife Research Unit, Department of Natural Resource Ecology and Management, Oklahoma State University, Stillwater, Oklahoma, USA. ORCID: 0000-0002-8658-9949

* Corresponding author
E-mail: Andrew.Taylor@ung.edu (ATT)

Supplemental Figures

Any use of trade, firm, or product names is for descriptive purposes only and does not imply endorsement by the U.S. Government.

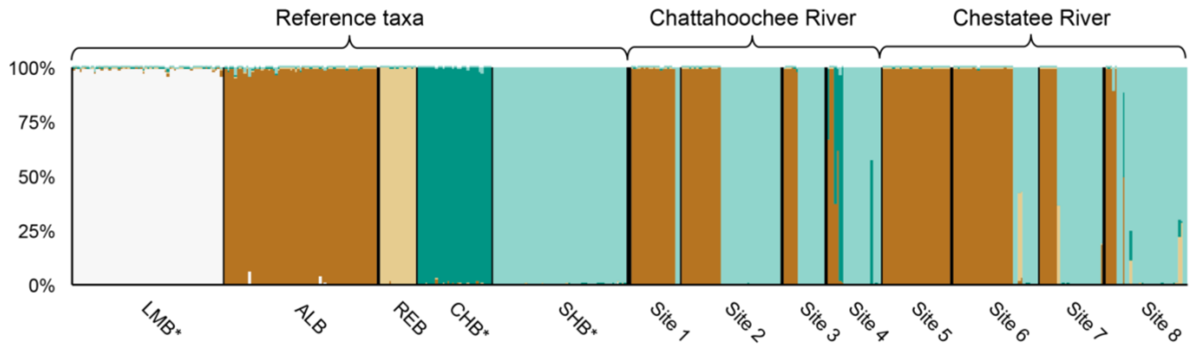

Fig S1. Individual STRUCTURE proportional assignments (*q*) for *Case Study I* (Lake Lanier, Georgia), ordered by site as in the main text, but also showing the reference specimens included for each taxon during this analysis. Please note that the colors used to denote clusters in this figure do not correspond to the palettes adopted in the main manuscript. Asterisk (*) indicates taxon is considered native to the study system. Non-native Alabama Bass (ALB), native Chattahoochee Bass (CHB), native Largemouth Bass (LMB), previously undocumented Redeye Bass (REB), and native Shoal Bass (SHB).

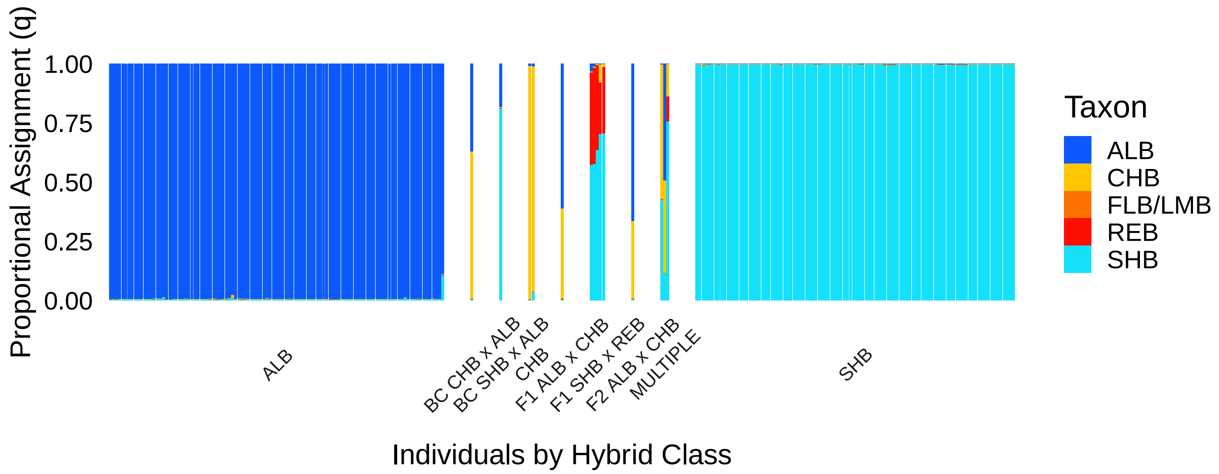

Fig S2. Individual STRUCTURE proportional assignments (*q*) for *Case Study I* (Lake Lanier, Georgia), ordered by hybrid classification in NEWHYBRIDS. Cluster colors correspond to the following black bass taxa: non-native Alabama Bass (ALB), native Chattahoochee Bass (CHB), native Florida Bass or Largemouth Bass (FLB/LMB), previously undocumented Redeye Bass (REB), and native Shoal Bass (SHB). Backcross to parental species (BC), first filial hybrid (F1), second filial hybrid (F2).

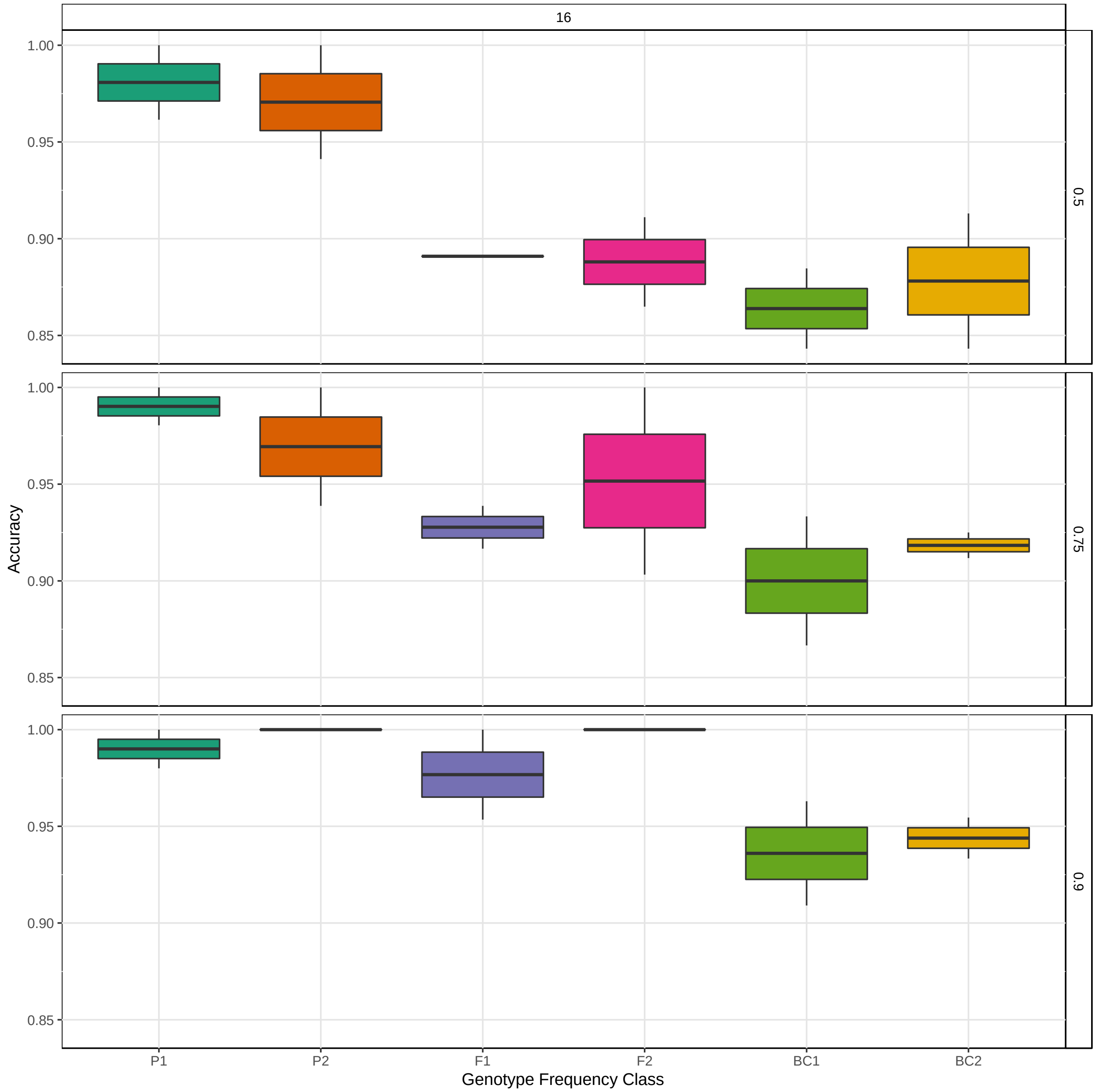

Fig S3. Results from ‘hybriddetective’ simulations mixing native Shoal Bass (P1) and non-native Alabama Bass (P2). Boxplots display accuracy of assignment to each hybrid class at three different critical posterior probability thresholds (0.50, 0.75, and 0.90). Parental species one (P1), parental species two (P2), first filial hybrid (F1), second filial hybrid (F2), backcross to parental species one (BC1), and backcross to parental species two (BC2).

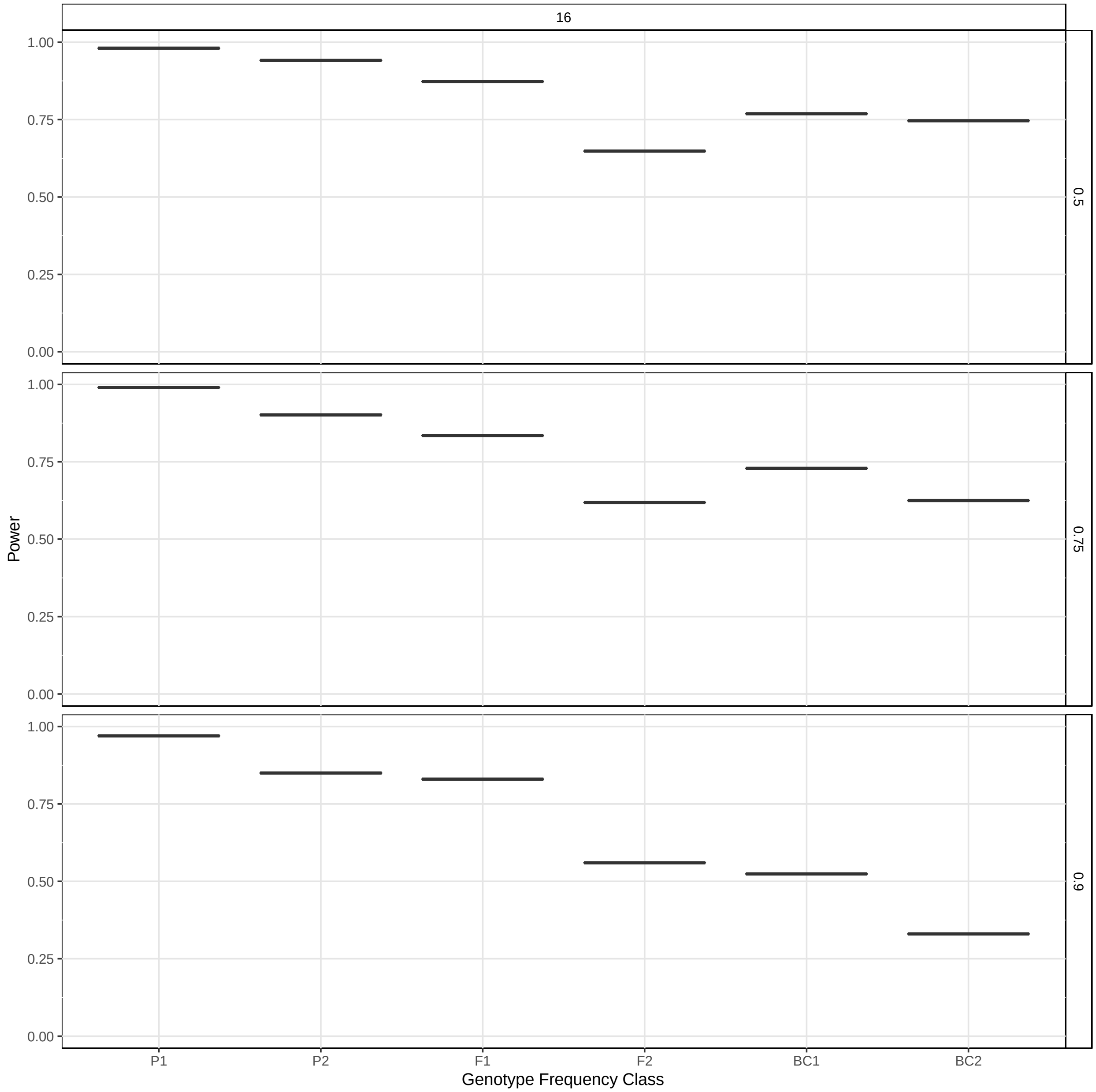

Fig S4. Results from 'hybriddetective' simulations mixing native Shoal Bass (P1) and non-native Alabama Bass (P2). Boxplots display power of assignment to each hybrid class at three different critical posterior probability thresholds (0.50, 0.75, and 0.90). Parental species one (P1), parental species two (P2), first filial hybrid (F1), second filial hybrid (F2), backcross to parental species one (BC1), and backcross to parental species two (BC2).

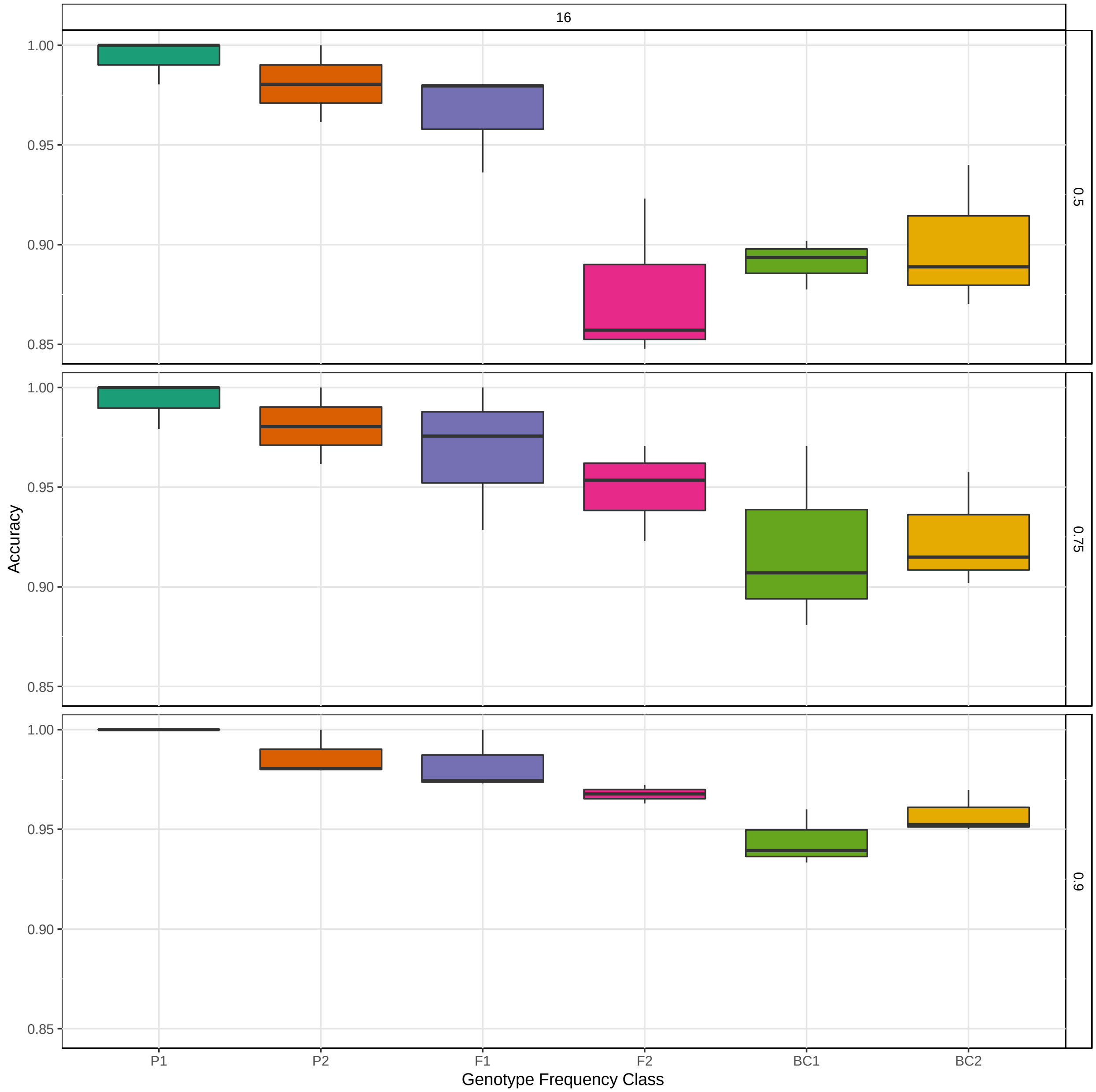

Fig S5. Results from 'hybriddetective' simulations mixing native Chattahoochee Bass (P1) and non-native Alabama Bass (P2). Boxplots display accuracy of assignment to each hybrid class at three different critical posterior probability thresholds (0.50, 0.75, and 0.90). Parental species one (P1), parental species two (P2), first filial hybrid (F1), second filial hybrid (F2), backcross to parental species one (BC1), and backcross to parental species two (BC2).

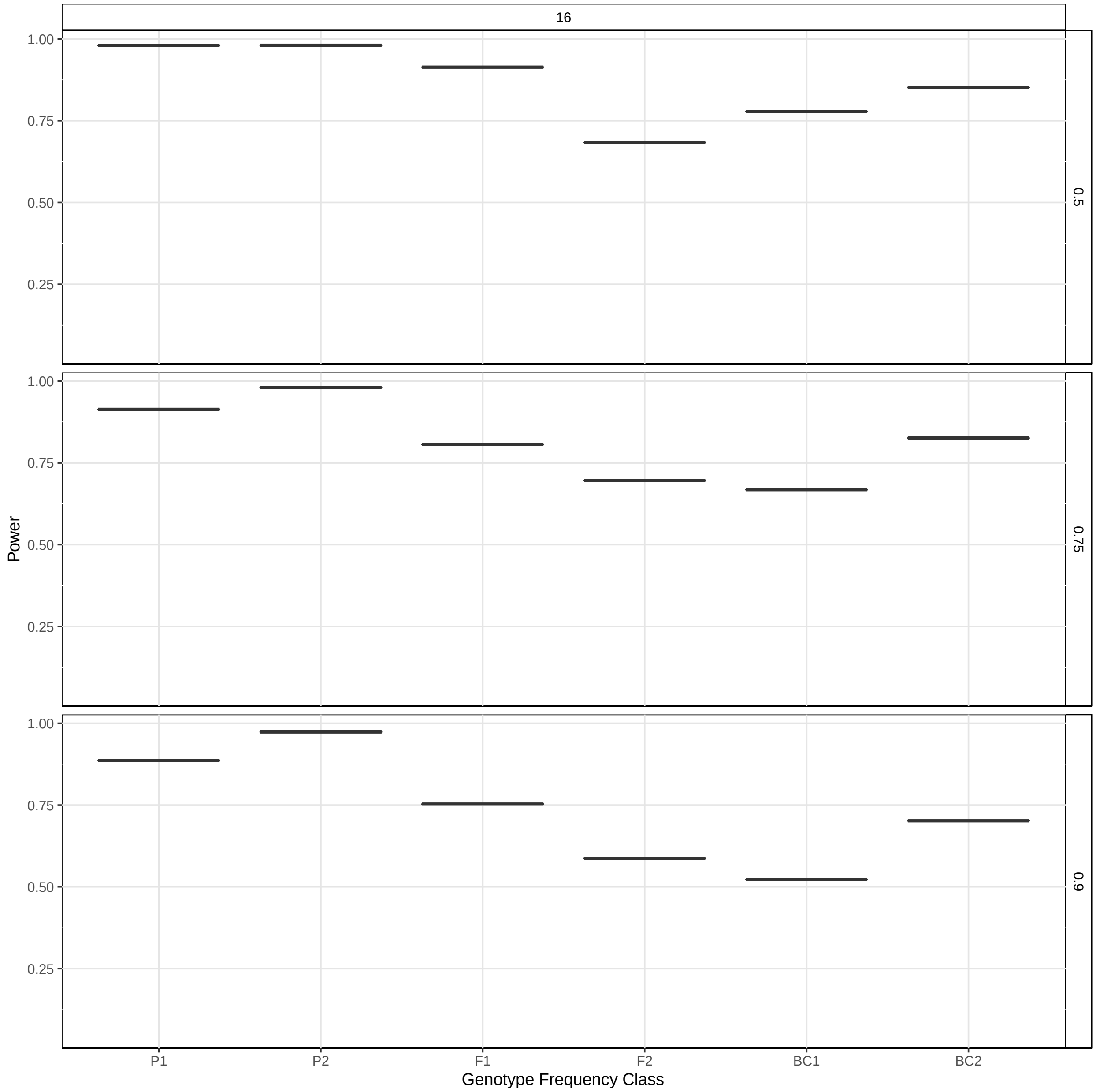

Fig S6. Results from 'hybriddetective' simulations mixing native Chattahoochee Bass (P1) and non-native Alabama Bass (P2). Boxplots display power of assignment to each hybrid class at three different critical posterior probability thresholds (0.50, 0.75, and 0.90). Parental species one (P1), parental species two (P2), first filial hybrid (F1), second filial hybrid (F2), backcross to parental species one (BC1), and backcross to parental species two (BC2).

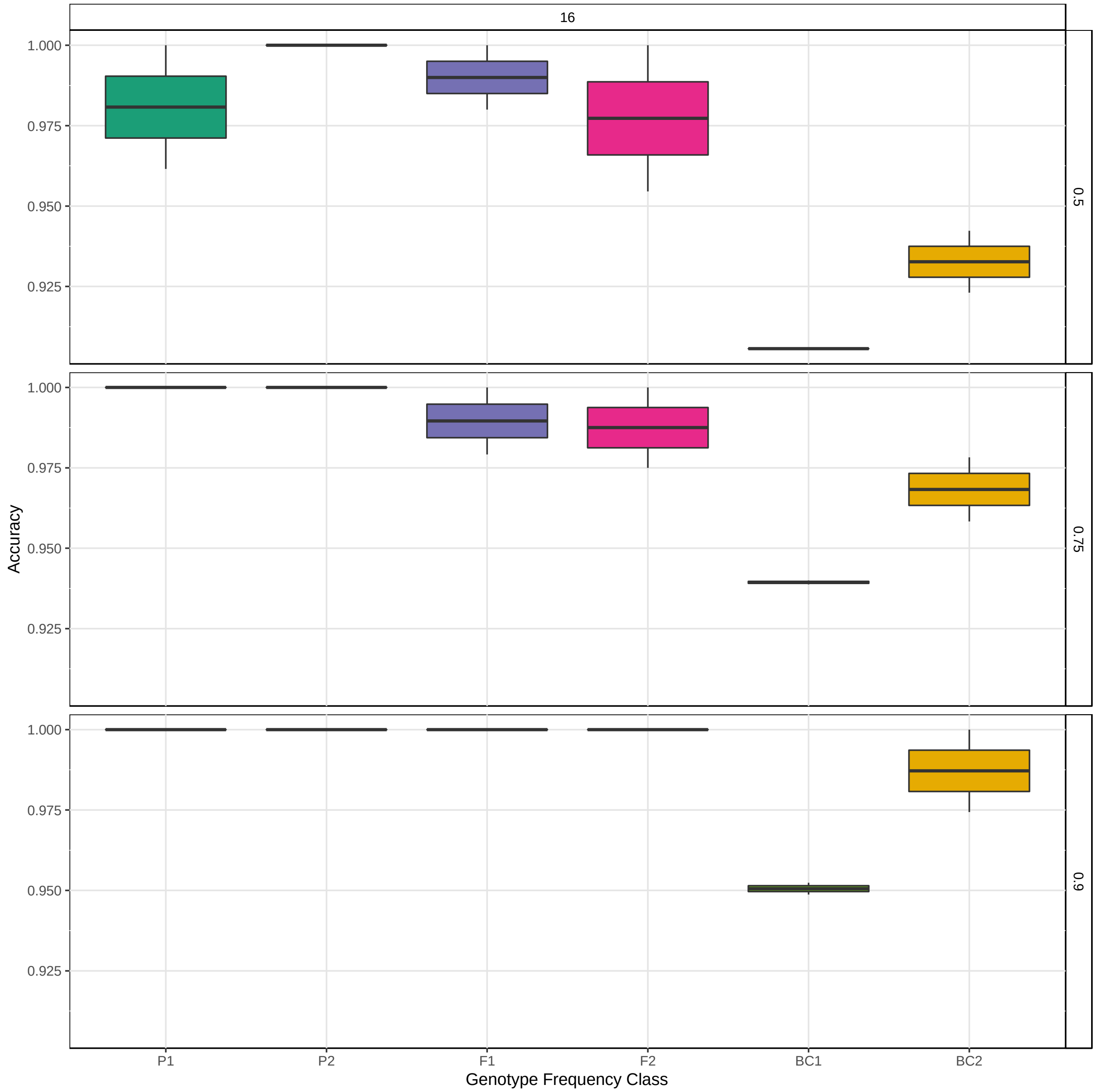

Fig S7. Results from 'hybriddetective' simulations mixing native Shoal Bass (P1) and previously undocumented Redeye Bass (P2). Boxplots display accuracy of assignment to each hybrid class at three different critical posterior probability thresholds (0.50, 0.75, and 0.90). Parental species one (P1), parental species two (P2), first filial hybrid (F1), second filial hybrid (F2), backcross to parental species one (BC1), and backcross to parental species two (BC2).

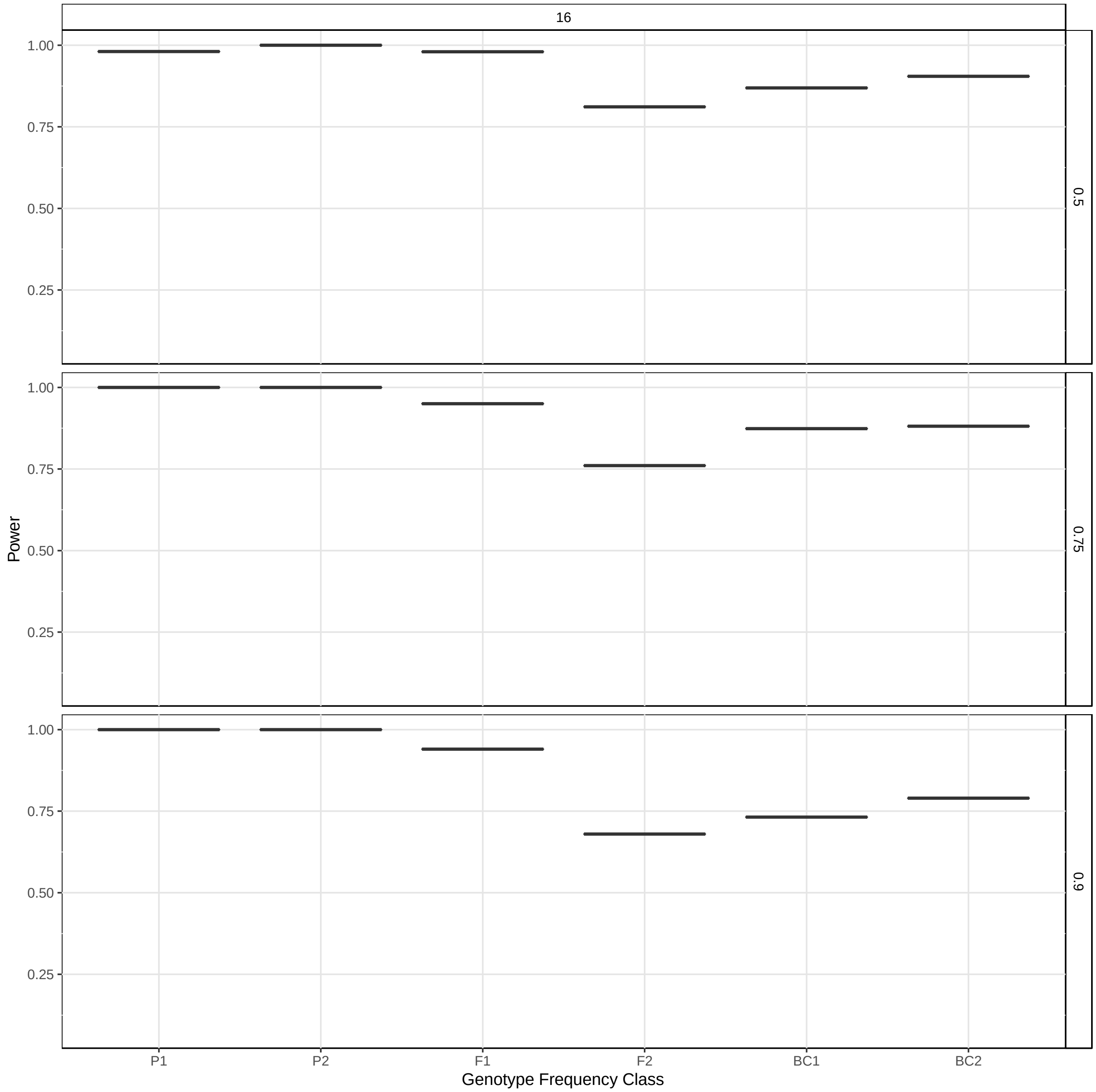

Fig S8. Results from 'hybriddetective' simulations mixing native Shoal Bass (P1) and previously undocumented Redeye Bass (P2). Boxplots display power of assignment to each hybrid class at three different critical posterior probability thresholds (0.50, 0.75, and 0.90). Parental species one (P1), parental species two (P2), first filial hybrid (F1), second filial hybrid (F2), backcross to parental species one (BC1), and backcross to parental species two (BC2).

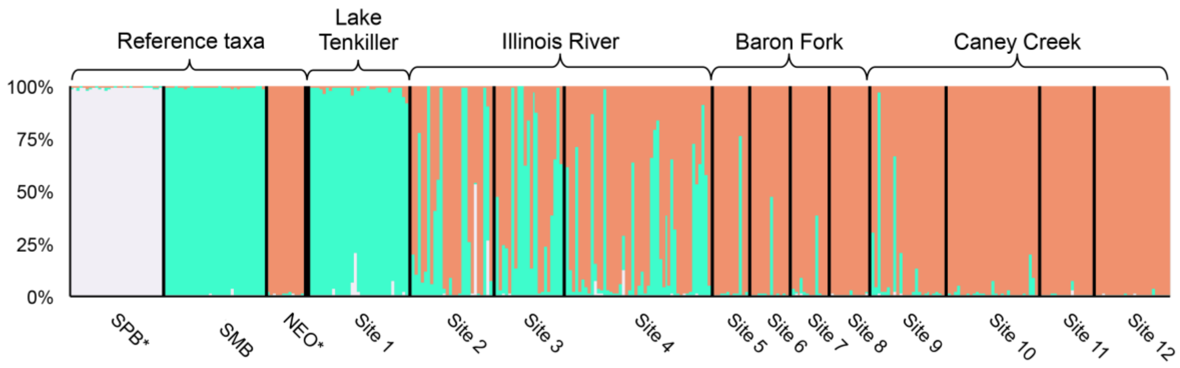

Fig S9. Individual STRUCTURE proportional assignments (*q*) for *Case Study II*, ordered by site as in the main text, but also showing the reference specimens included for each taxon during this analysis. Please note that the colors used to denote clusters in this figure do not correspond to the palettes adopted in the main manuscript. Asterisk (*) indicates taxon is considered native to the study system. Native Neosho Bass (NEO), non-native Smallmouth Bass (SMB), native Spotted Bass (SPB).

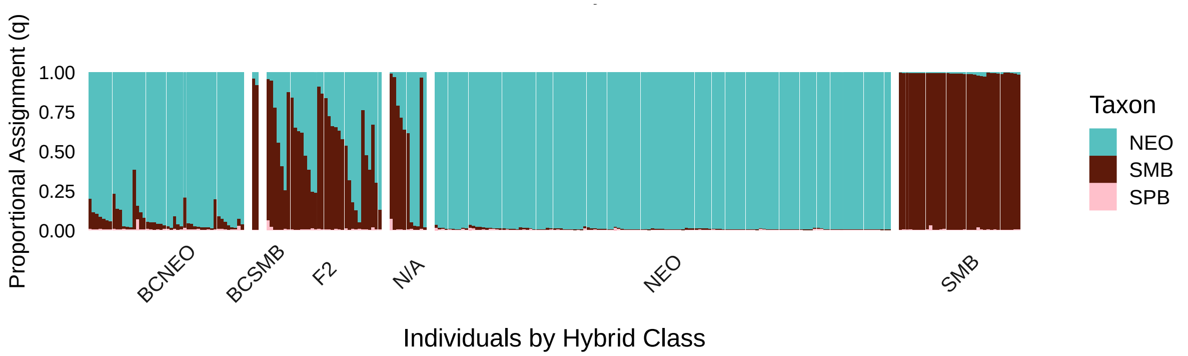

Fig S10. Individual STRUCTURE proportional assignments (q) for *Case Study II* (Lake Tenkiller, Oklahoma), ordered by hybrid classification in NEWHYBRIDS. Cluster colors correspond to native Neosho Bass (NEO), non-native Smallmouth Bass (SMB), and native Spotted Bass (SPB). Note n=11 fish with an assignment of "N/A" were not classified as their NEWHYBRIDS P of Z were all below the applied 0.50 critical threshold for classification. Backcross to parental species (BC), first filial hybrid (F1), second filial hybrid (F2).

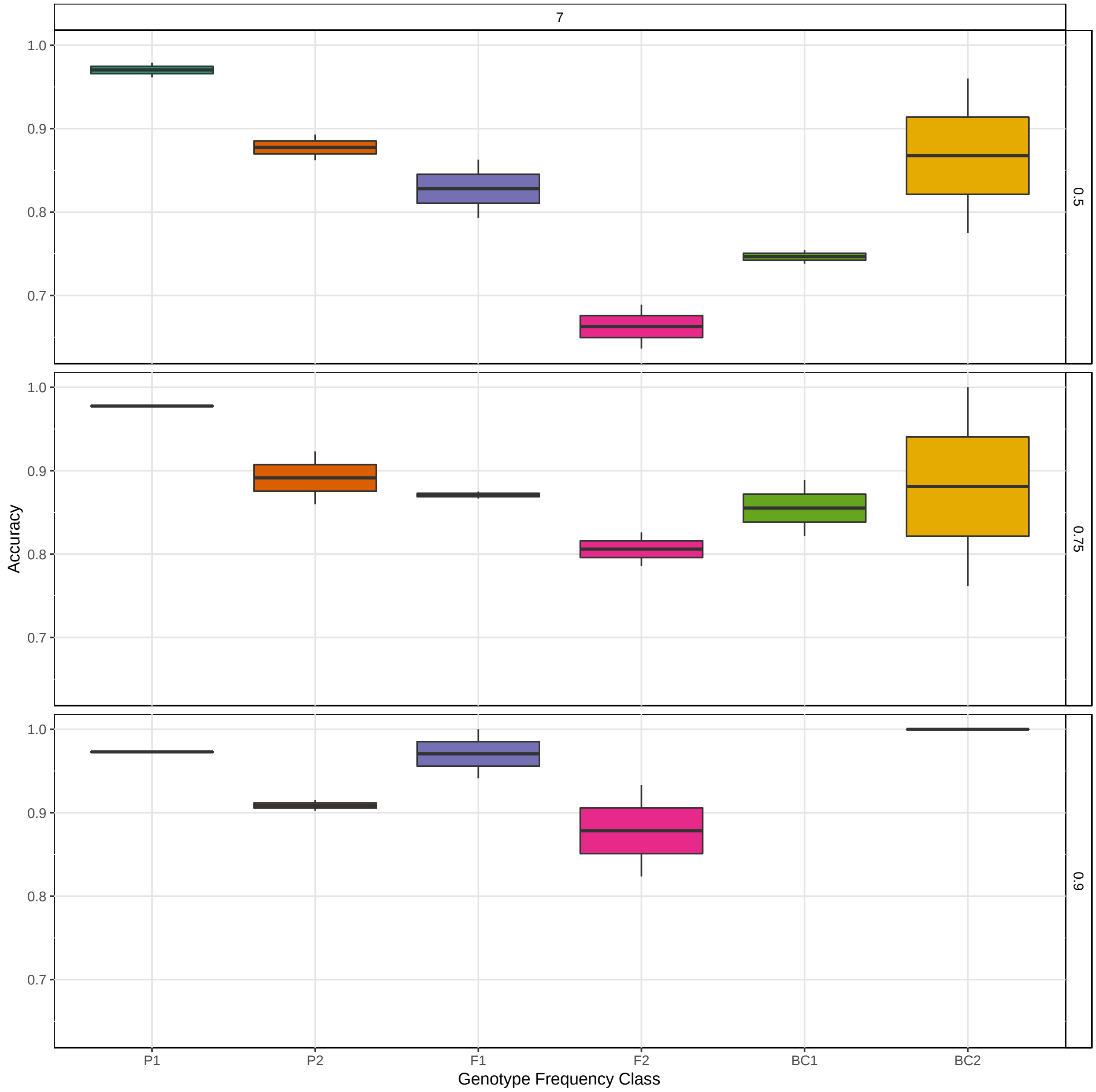

Fig S11. Results from 'hybriddetective' simulations mixing native Neosho Bass (P1) and non-native Smallmouth Bass (P2). Boxplots display accuracy of assignment to each hybrid class at three different critical posterior probability thresholds (0.50, 0.75, and 0.90). Parental species one (P1), parental species two (P2), first filial hybrid (F1), second filial hybrid (F2), backcross to parental species one (BC1), and backcross to parental species two (BC2).

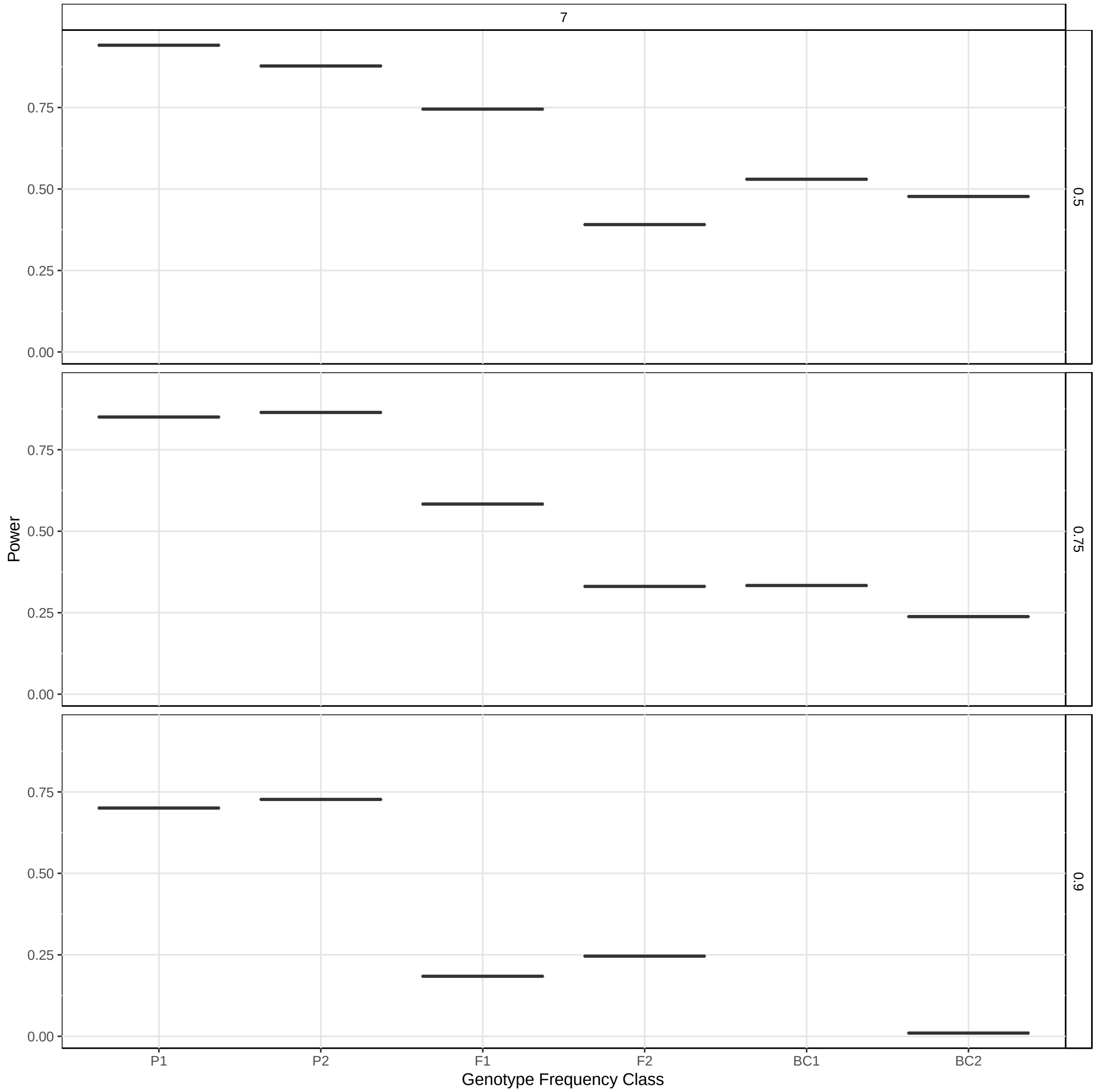

Fig S12. Results from 'hybriddetective' simulations mixing native Neosho Bass (P1) and non-native Smallmouth Bass (P2). Boxplots display power of assignment to each hybrid class at three different critical posterior probability thresholds (0.50, 0.75, and 0.90). Parental species one (P1), parental species two (P2), first filial hybrid (F1), second filial hybrid (F2), backcross to parental species one (BC1), and backcross to parental species two (BC2).
