# Supplementary material for: Impoundments facilitate upstream invasion and introgression: Case studies of fluvial black basses (Micropterus spp.) in the southeastern USA
Source: PLoS One. 2025 Feb 5;20(2):e0315620. doi: 10.1371/journal.pone.0315620 (PMC11798496; doi:10.1371/journal.pone.0315620)
Supplement: S2 File — (PDF) [file pone.0315620.s004.pdf]

Impoundments facilitate upstream invasion and introgression:
Case studies of fluvial black basses (*Micropterus* spp.) in the southeastern USA

Andrew T. Taylor[1,2]*, Michael D. Tringali[3], and James M. Long[4]

[1] Department of Natural Resource Ecology and Management, Oklahoma State University, Stillwater, Oklahoma, USA. ORCID: 0000-0002-8491-9967

[2] Department of Biology, University of North Georgia, Dahlonega, Georgia, USA.

[3] Florida Fish and Wildlife Conservation Commission, Fish and Wildlife Research Institute, St. Petersburg, Florida, USA. ORCID: 0000-0002-9336-9207

[4] U.S. Geological Survey, Oklahoma Cooperative Fish and Wildlife Research Unit, Department of Natural Resource Ecology and Management, Oklahoma State University, Stillwater, Oklahoma, USA. ORCID: 0000-0002-8658-9949

* Corresponding author
E-mail: Andrew.Taylor@ung.edu (ATT)

Supplemental Tables

Any use of trade, firm, or product names is for descriptive purposes only and does not imply endorsement by the U.S. Government.

Table S1. Sample site information for *Case Study I* (Lake Lanier, Georgia). Table includes sample site numbers (corresponding to Fig 1A), stream names, site names, geospatial coordinates in decimal degrees, years that collections were made, approximate elevation of each site (m), approximate number of river-kilometers (rkm) of each site from river-impoundment interface ("RII"), and the number of black bass (*Micropterus* spp.) genotypes collected from each site.

Site #	Stream Name	Site Name	Latitude	Longitude	Elevation (m)	rkm from RII	Collection Year(s)	Genotypes Collected
1	Chattahoochee River	Flat Rock	34.466399	-83.686461	330	6.4	2013, 2014	20
2	Chattahoochee River	Bull Shoals	34.482440	-83.680216	330	8.8	2013, 2014	41
3	Chattahoochee River	Crow Island	34.503651	-83.666475	334	11.7	2013, 2014	17
4	Chattahoochee River	Buck Shoals	34.563347	-83.628713	362	24.0	2013, 2014	22
5	Chestatee River	Big Rock	34.458609	-83.966767	328	3.0	2013, 2014	28
6	Chestatee River	Canoe Launch	34.471844	-83.979555	330	5.8	2013, 2014	36
7	Chestatee River	Horseshoe Bend	34.492659	-83.997084	333	9.3	2013, 2014	26
8	Chestatee River	D.S. Hwy 60 bridge	34.504223	-83.968851	338	14.6	2013, 2014	35

D.S., downstream.

Table S2. Sample site information for *Case Study II* (Lake Tenkiller, Oklahoma). Table includes sample site numbers (corresponding to Fig 1B), stream names, site names, geospatial coordinates in decimal degrees, years that collections were made, approximate elevation of each site (m), approximate number of river-kilometers (rkm) of each site from river-impoundment interface ("RII"), and the number of black bass (*Micropterus* spp.) genotypes collected from each site.

Site #	Stream Name	Site Name	Latitude	Longitude	Elevation (m)	rkm from RII	Collection Year(s)	Genotypes Collected
1	N/A	Lake Tenkiller	35.59885	-95.044454	192	N/A	2014	32
2	Illinois River	U.S. of interface	35.842261	-94.920055	194	1.3	2015	26
3	Illinois River	Riverside Park	35.922055	-94.923975	203	12.7	2015	22
4	Illinois River	Round Hollow to Peavine	36.09421	-94.830422	240	54.0	2015	47
5	Baron Fork	Welling Rd bridge	35.870224	-94.896924	200	5.3	2015	11
6	Baron Fork	U.S. of Willow Branch Crk	35.894349	-94.863118	208	10.8	2015	12
7	Baron Fork	West of N 4580 Rd	35.912631	-94.846221	217	15.1	2015	12
8	Baron Fork	U.S. of Hwy 51 bridge	35.936556	-94.827673	220	19.1	2015	12
9	Caney Creek	D.S. of S 581 Rd access	35.793278	-94.846425	200	1.8	2015	24
10	Caney Creek	U.S. of S 581 Rd access	35.798125	-94.840462	210	2.8	2015	29
11	Caney Creek	Bidding Creek confl.	35.841145	-94.789427	229	10.9	2015	17
12	Caney Creek	N 4630 Rd crossing	35.841508	-94.77270	237	13.3	2015	24

D.S., downstream; U.S., upstream; confl., confluence.
